# The Combination of R848 with Sorafenib Enhances Antitumor Effects by Reprogramming the Tumor Immune Microenvironment and Facilitating Vascular Normalization in Hepatocellular Carcinoma

**DOI:** 10.1002/advs.202207650

**Published:** 2023-04-21

**Authors:** Yuchao He, Linlin Zhan, Jian Shi, Manyu Xiao, Ran Zuo, Chengmeng Wang, Zhiyong Liu, Wenchen Gong, Liwei Chen, Yi Luo, Shaojun Zhang, Youwei Wang, Lu Chen, Hua Guo

**Affiliations:** ^1^ Department of Tumor Cell Biology Tianjin Medical University Cancer Institute and Hospital Tianjin 300060 China; ^2^ National Clinical Research Center for Cancer Key Laboratory of Cancer Prevention and Therapy Tianjin's Clinical Research Center for Cancer Tianjin 300060 China; ^3^ Institute of Precision Medicine The First Affiliated Hospital Sun Yat‐sen University Guangzhou 510080 China; ^4^ School of Pharmacy Minzu University of China Beijing 10081 China; ^5^ Department of Pathology Tianjin Medical University Cancer Institute and Hospital Tianjin 300060 China; ^6^ Medical Research Institute Guangdong Provincial People's Hospital Guangdong Academy of Medical Sciences Guangzhou 510080 China; ^7^ Institute of Medical Engineering & Translational Medicine Tianjin University Tianjin 300072 China; ^8^ Department of Hepatobiliary Cancer Liver Cancer Research Center Tianjin Medical University Cancer Institute and Hospital Tianjin 300060 China

**Keywords:** hepatocellular carcinoma, immune microenvironment, R848, sorafenib, vascular normalization

## Abstract

Novel promising strategies for combination with sorafenib are urgently needed to enhance its clinical benefit and overcome toxicity in hepatocellular carcinoma (HCC). the molecular and immunomodulatory antitumor effects of sorafenib alone and in combination with the new immunotherapeutic agent R848 are presented. Syngeneic HCC mouse model is presented to explore the antitumor effect and safety of three sorafenib doses alone, R848 alone, or their combination in vivo. R848 significantly enhances the sorafenib antitumor activity at a low subclinical dose with no obvious toxic side effects. Furthermore, the combination therapy reprograms the tumor immune microenvironment by increasing antitumor macrophages and neutrophils and preventing immunosuppressive signaling. Combination treatment promotes classical M1 macrophage‐to‐FTH1^high^ M1 macrophage transition. The close interaction between neutrophils/classical M1 macrophages and dendritic cells promotes tumor antigen presentation to T cells, inducing cytotoxic CD8^+^ T cell‐mediated antitumor immunity. Additionally, low‐dose sorafenib, alone or combined with R848, normalizes the tumor vasculature, generating a positive feedback loop to support the antitumor immune environment. Therefore, the combination therapy reprograms the HCC immune microenvironment and normalizes the vasculature, improving the therapeutic benefit of low‐dose sorafenib and minimizing toxicity, suggesting a promising novel immunotherapy (R848) and targeted therapy (tyrosine kinase inhibitors) combination strategy for HCC treatment.

## Graphical abstract

Combined treatment with low‐dose sorafenib and R848 reprograms the tumor immune microenvironment by increasing the population and activation of antitumor macrophages and neutrophils. These cells later activate dendritic cells, thus resulting in more efficient antigen presentation to T cells. TNF‐*α* secreted by T cells contributes to vascular normalization, which in turn supports immune cell infiltration, forming a positive feedback loop.

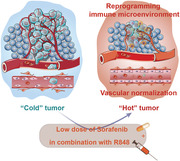

## 1 Introduction

Hepatocellular carcinoma (HCC) is the third leading cause of cancer‐related death worldwide.^[^ ^1^, ^2^ ^]^ Most HCC patients are diagnosed at advanced stages, missing the window for surgical resection, which results in limited therapeutic options and a poor prognosis.^[^ ^3^ ^]^ Sorafenib, a broad‐spectrum tyrosine kinase inhibitor (TKI) with potent antiangiogenic effects mediated by targeting VEGFR1‐3 and PDGFR, has been the standard first‐line systemic therapy for advanced HCC for more than 10 years.^[^ ^4^, ^5^ ^]^ Until 2018, another approved first‐line targeted therapy, lenvatinib, showed non‐inferior results compared to those of sorafenib.^[^ ^6^ ^]^ Unfortunately, the clinical response rate to sorafenib is less than 20%, and the overall survival (OS) benefits are limited by primary or secondary resistance.^[^ ^7^, ^8^ ^]^ This is probably because sorafenib‐mediated antiangiogenic effects cause the excessive pruning of vessels and increase hypoxia, resulting in the generation of an immunosuppressive tumor microenvironment (TME) characterized by the intratumoral accumulation of regulatory T cells (Tregs) and M2 macrophages and the upregulation of programmed death ligand 1 (PD‐L1) expression, which eventually results in evasive resistance.^[^ ^9^, ^10^, ^11^ ^]^ Therefore, activation of the immune response and maintenance of the physiological composition of the immune system might improve the clinical efficacy of sorafenib.

Recently, immunotherapy with immune checkpoint blockade (ICB) agents targeting cytotoxic T lymphocyte‐associated antigen 4, programmed cell death protein 1 (PD‐1), or PD‐L1 has revolutionized the clinical management of various tumors and has shown potential clinical advantages, sparking great interest in its application in HCC.^[^ ^12^ ^]^ Nevertheless, only 15–20% of HCC patients respond to these treatments,^[^ ^13^ ^]^ and 20–50% of patients with advanced HCC receiving anti‐PD‐1/PD‐L1 monotherapy experience severe immune‐related adverse events,^[^ ^14^ ^]^ especially hepatotoxicity induced by aberrantly activated monocytes and a shift in T cell effector functions.^[^ ^15^ ^]^ The limitations of monotherapy‐based approaches have raised interest in the development of combinations of immunotherapy and targeted therapies for the treatment of HCC. Currently, some combination therapies including TKIs, such as lenvatinib or apatinib, with an ICB agent have shown benefits for patients with advanced HCC.^[^ ^16^, ^17^ ^]^ However, combination treatment with an anti‐PD‐1 antibody and sorafenib failed in mouse model experiments and showed no additional antitumor activity.^[^ ^9^ ^]^ A high‐profile phase III clinical trial of lenvatinib combined with an anti‐PD‐1 antibody (NCT03713593) failed to meet the primary endpoints for OS and progression‐free survival. These results suggest that there is still a need to explore optimal drug combinations. In addition, the proportion of patients with severe adverse events after combination therapy was demonstrated to be as high as 67%, owing to the additive and mostly non‐overlapping toxicities of anti‐PD‐1/PD‐L1 antibodies and antiangiogenic agents.^[^ ^16^ ^]^ Therefore, an exploration of novel promising strategies for combined use with sorafenib is urgently needed to overcome drug resistance and enhance clinical benefits while minimizing toxicity.

R848, a Toll‐like receptor 7/8 (TLR7/8) agonist, is a promising agent that can remodel the tumor immune microenvironment through TLR signaling to enhance antitumor effects. Given that TLRs are expressed by multiple immune cells, including macrophages, dendritic cells (DCs), neutrophils, B cells, and natural killer cells, TLR7/8 stimulation can not only control the activation, maturation, and immunological functions of myeloid cells, but also augment the activity of adaptive immunity. Indeed, TLR7/8 agonists, including imiquimod and its more potent counterpart resiquimod (R848), have been approved by the Food and Drug Administration (FDA) for dermatologic malignancies, including superficial basal cell carcinoma.^[^ ^18^ ^]^ Moreover, the success of R848 as an immunostimulatory agent has previously been demonstrated, showing good safety and efficacy in a variety of preclinical anticancer immunotherapy models and clinical trials.^[^ ^19^, ^20^, ^21^ ^]^ (NCT00960752, NCT04127864, NCT01094496, and NCT04799054) Notably, R848 can promote cytotoxic T lymphocyte and IL‐12 production and induce HCC tumor‐specific immunity when combined with HMGN1.^[^ ^22^ ^]^ However, whether it can improve the immune microenvironment and enhance the clinical efficacy of sorafenib remains unknown. In particular, considering the different modes of action of immunotherapeutics (microenvironment‐targeted) and sorafenib (tumor cell‐targeted and anti‐angiogenic), it is necessary to elucidate the potential efficacy of combined treatment with R848 and sorafenib for HCC.

Previous studies have shown that antiangiogenic drugs have a “normalization window,” depending on the time and dose.^[^ ^23^, ^24^ ^]^ The TME induced by high‐dose antiangiogenic treatment, which is characterized by a low oxygen level, low pH, and abnormal vessels, can reduce the effectiveness of immunotherapy.^[^ ^11^ ^]^ A recent study also found reciprocal mediating effects between vascular normalization and immune responses.^[^ ^25^ ^]^ Briefly, vessel normalization lead to better perfusion of oxygen and nutrients to the tumor, which can help reduce hypoxia and vascular leakage and improve the delivery of immune cells and anticancer drugs, allowing for more efficient immune cell infiltration and enhancing the response to immune therapy.^[^ ^24^, ^26^ ^]^ Conversely, immune cells can also secrete factors that promote vessel normalization, such as IFN‐*γ* secreted by activated T cells^[^ ^27^ ^]^ and IL‐12 and TNF‐*α* secreted by M1 macrophages,^[^ ^28^ ^]^ ultimately forming a positive feedback loop favorable for the establishment of an antitumor immune microenvironment. In the present study, we combined R848 with different doses of sorafenib to treat HCC. The anticancer activity was significantly enhanced when R848 was combined with low‐dose sorafenib, and little toxicity was observed. Here, we further hypothesized that combining R848 with low‐dose sorafenib would extend the window of normalization and enhance the antitumor immune response. Normalization of the TME facilitated R848‐mediated remodeling of the immune composition, mainly by increasing the numbers of macrophages and neutrophils and reducing those of Tregs. In addition, the combination therapy promoted the repolarization of classical M1 macrophages into cytotoxic FTH1^high^ M1 macrophages, but not M2 macrophages, and also activated macrophages and neutrophils to interact with and recruit DCs, which presented more tumor‐associated antigens (TAAs) to cytotoxic CD8 T^+^ cells to enhance tumor cell killing. These data provide evidence for the potential clinical application of R848 in combination with low‐dose sorafenib as HCC therapy.

## 2 Results

### 2.1 Combination Therapy with R848 and Low‐Dose Sorafenib Significantly Increases Antitumor Effects with Few Toxic Side Effects in HCC

**Figure 1:**
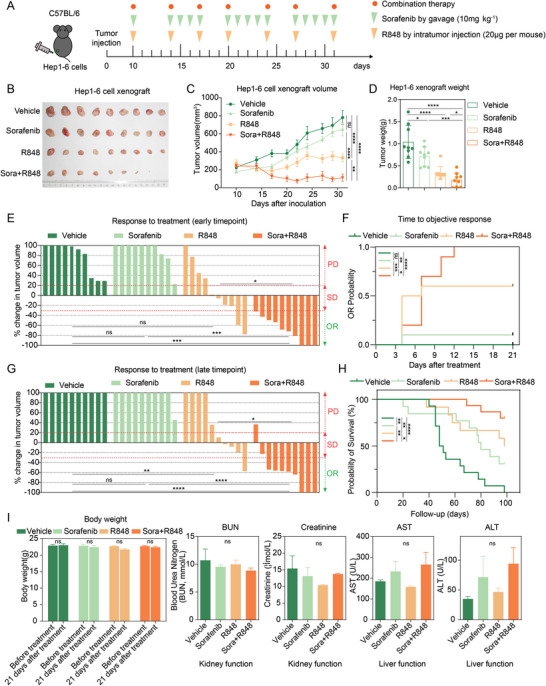
Combination therapy with R848 and low‐dose sorafenib (10 mg kg^−1^) significantly increases antitumor activity and prolongs mouse survival, with few toxic side effects, in the syngeneic Hepa1‐6 model. A) The timeline and schedule of procedures in the Hepa1‐6 subcutaneous tumor‐bearing model. Hepa1‐6 mouse HCC cells were subcutaneously injected into C57BL/6 mice. When the tumor volume reached ≈200 mm^3^, the mice were treated with sorafenib (10 mg kg^−1^) intragastrically, with R848 (20 µg per mouse), with sorafenib combined with R848, or with vehicle (*n* = 9 mice per group) until they met the treatment endpoint. B) Representative photographs of subcutaneous Hepa1‐6 HCC tumors after the indicated treatments. C) Growth curve of Hepa1‐6 tumors at the indicated time points. D) Tumor weight at the endpoint after the indicated treatments. E) Response to treatment at the early time point on the 21st day (*n* = 10 mice per group). The difference in the PD rate is indicated by the upper part, while the difference in the ORR is indicated by the lower part. F) The time to objective response in mice after the indicated treatments. G) Response to treatment at the late time point on the 31st day (*n* = 10 mice per group). H) Kaplan‒Meier survival curves of treated mice (vehicle, *n* = 14; sorafenib, *n* = 13; R848, *n* = 12; sorafenib+R848, *n* = 15). I) The effects of sorafenib and/or R848 treatment on body weight and kidney and liver function in mice bearing Hepa1‐6 xenografts after treatment for 21 days. Serum levels of BUN, creatinine, AST, and ALT were measured in all treatment groups after the experiment (*n* = 9 mice per group). PD, progressive disease. SD, stable disease. OR, objective response. AST, aspartate aminotransferase. ALT, alanine transaminase. The error bars indicate the means ± SEMs; ns: *p* > 0.05, * *p* < 0.05, ** *p* < 0.01, *** *p* < 0.001, **** *p* < 0.0001, one‐way ANOVA C–G,I), log‐rank test H).

To explore whether combination therapy with R848 and sorafenib could improve the antitumor effect on HCC, we used a subcutaneous syngeneic HCC mouse model (**Figure** **1**A). We treated mice with an established HCC tumor (≈200 mm^3^ volume) with sorafenib at three different doses via gavage (clinical standard dose, 30 mg kg^−1^; low subclinical dose, 10 mg kg^−1^; and lowest dose, 3 mg kg^−1^), R848 (20 µg per mouse via intratumoral injection), or their combination (Figure 1A; and Figures S1A and S2A, Supporting Information). After a subsequent evaluation of dosing in vivo, we found that although tumor growth was efficiently suppressed with 30 mg kg^−1^ of sorafenib and R848 (Figure S1B, Supporting Information), the combination therapy led to body weight loss on the 16th day (Figure S1C,D, Supporting Information) and apparently reduced the survival rates (4/10 on day 16; 1/10 on day 21) compared to those with the other treatments (Figure S1A,E, Supporting Information), suggesting toxicity and side effects. In contrast, the combination of the lowest dose of sorafenib (3 mg kg^−1^) with R848 resulted in compromised antitumor effects that were not superior to those achieved with drug alone (Figure S2B–D, Supporting Information), but no changes were observed in body weight or survival rates (Figure S2A,E, Supporting Information).

Notably, combination therapy with a low subclinical dose of sorafenib (10 mg kg^−1^) and R848 produced a stronger synergistic effect with significant tumor regression compared to that with either drug alone or the solvent control (vehicle group). As indicated by the tumor growth curves, 10 mg kg^−1^ sorafenib exerted slight tumor‐suppressive effects. R848 exhibited relatively good inhibitory effects, but the tumors maintained their growth trend. Remarkably, the tumor volumes in the combined group showed a significant reduction after only two cycles of administration, even though the tumors of two mice completely disappeared by day 22 and did not recur after 5 weeks (Figure 1B,C). In addition, a significant decrease in tumor weight was observed in the groups treated with low‐dose sorafenib and R848 (combination 0.179 ± 0.149 g vs vehicle 1.038 ± 0.354 g, *p* < 0.0001; vs sorafenib 0.712 ± 0.203 g, *p* < 0.001; and vs R848 0.336 ± 0.139 g, *p* < 0.05) (Figure 1D).

Notably, the combination therapy achieved a significantly better objective response rate (ORR) than either sorafenib or R848 alone at the early (21 days) and late (31 days) time points. In contrast, all mice receiving a low dose of sorafenib (10 mg kg^−1^) had progressive disease, and mice treated with R848 achieved a significantly higher ORR than those treated with vehicle only at late time points (Figure 1E,G). Furthermore, combination‐treated mice required less time to achieve an objective response (Figure 1F) and had a better OS rate than the vehicle‐ and sorafenib‐treated mice (Figure 1H). As expected, 10 mg kg^−1^ sorafenib, R848, or their combination showed no overt systemic toxicity, as no significant changes in body weight or blood urea nitrogen /creatinine and aspartate aminotransferase/alanine aminotransferase levels were observed, suggesting normal kidney and liver functions (Figure 1I). Thus, the results indicated that the combination of 10 mg kg^−1^ sorafenib and R848 not only maintained optimal antitumor efficacy, but also ensured safety and minimal toxicity. It is necessary to clarify that the dose used in our study in mice (30 mg kg^−1^ day^−1^) is equivalent to the clinically recommended dose and consistent with the dose reported in a previous study.^[^ ^29^ ^]^ Further, the gavage administration mode of sorafenib in our study also simulates the clinical practice of oral administration. Considering the toxic effects of combination treatment with a clinical dose of 30 mg kg^−1^ sorafenib and R848 (Figure S1, Supporting Information) and the results shown in Figure 1, we selected 10 mg kg^−1^ sorafenib as the optimal dose for combination with R848 in this study. Overall, combination therapy with low‐dose sorafenib and R848 could improve the curative effect on HCC in mice with fewer toxic effects compared to that with no treatment or single‐drug treatment.

### 2.2 The Combination of R848 and Low‐Dose Sorafenib Reprograms the Tumor Immune Microenvironment and Prevents Sorafenib‐Induced Immunosuppression

**Figure 2:**
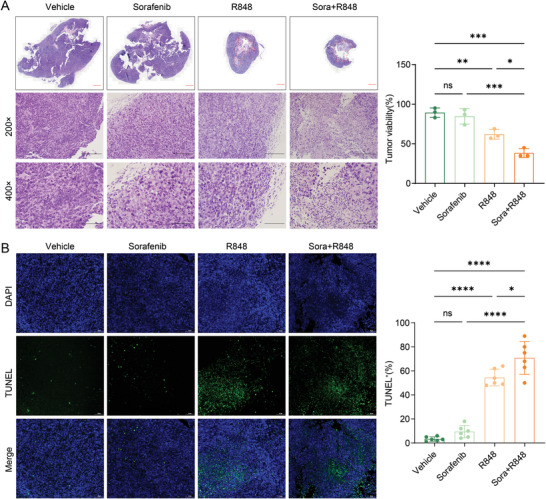
Combination therapy with R848 and low‐dose sorafenib significantly promotes tumor apoptosis and immune infiltration. A) The landscape and representative images of H&E staining of tumor tissues from four groups are shown (200× & 400×). Scale bars, 2000, 200, and 100 µm. The dashed box on the left shows the necrotic area of the tumor tissues after the indicated treatments, and the histogram on the right shows the percentage of viable tumor cells. B) TUNEL assay of tumor samples (40×). Representative staining images (left) and TUNEL‐positive cell rates (right) are shown. Scale bar, 100 µm. The error bars indicate the means ± SEMs; ns: *p* > 0.05, * *p* < 0.05, ** *p* < 0.01, *** *p* < 0.001, **** *p* < 0.0001, one‐way ANOVA.

Having reproduced the clinical benefit observed with a low dose of sorafenib combined with immunotherapy (R848) in a mouse model, we examined the mechanism of action related to the tumor immune microenvironment. We first utilized microdissected tumor tissues (MDTs) on a chip, an ex vivo method of drug testing and personalized therapy,^[^ ^30^ ^]^ to evaluate the response of 37 surgically resected HCC tissue samples to a standard clinical dose of sorafenib. Based on the analysis of the MDTs, these patients were divided into responder and nonresponder groups (*n* = 5 vs 32) (Figure S3A, Supporting Information). We then performed RNA sequencing (RNA‐seq) of the 37 HCC tissue samples and analyzed the immune composition of the tumors using the bulk tissue gene expression profile. Immunosuppressive cells, including Tregs and M2 macrophages, predominantly showed enrichment in patients who did not respond to the clinical dose of sorafenib (Figure S3B–D, Supporting Information). Previous studies have shown that prolonged or excessive doses of anti‐angiogenic therapy can lead to tissue hypoxia‐driven angiogenesis and immunosuppression.^[^ ^9^, ^31^, ^32^ ^]^ These findings suggest that the immunosuppressive microenvironment might have caused the limited efficacy of sorafenib and the associated evasive resistance. Interestingly, many inflammatory cells infiltrated the tumor tissue, and lower tumor viability was observed through hematoxylin and eosin (H&E) staining after combination treatment with low‐dose sorafenib and R848 compared to the other treatments (**Figure** **2**A). A TUNEL assay of the tumor tissues showed greater induction of apoptosis by the combination treatment than by treatment with vehicle or either drug alone (Figure 2B). Thus, we speculated that a low dose of sorafenib (10 mg kg^−1^) combined with R848 might promote immune cell infiltration to achieve better antitumor effects. These results prompted us to further explore the specific effects of combination therapy on immune activation.

**Figure 3:**
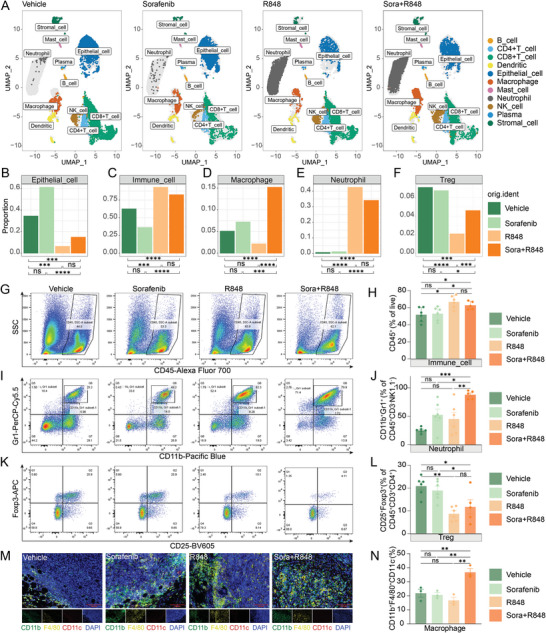
Combination treatment with R848 and low‐dose sorafenib alters the tumor microenvironment. A) Tumor tissues from the four groups after the indicated treatments were processed into single‐cell suspensions, and unsorted cells were used for 10× Genomics scRNA‐seq. The UMAP plots of tumor tissues showed 11 clusters, including clusters corresponding to major immune cell subtypes and tumor cells. Each cluster is presented in a different color based on the analysis of single‐cell transcriptome data. B–F) Bar plots showing the proportions of 5 major cell types in different tumor tissues after the indicated treatments: epithelial cells B), immune cells C), macrophages D), neutrophils E), and Tregs F). G–L) Flow cytometric analysis of tumor‐infiltrating immune cells in the Hepa1‐6 syngeneic mouse model after treatment with vehicle, sorafenib (10 mg kg^−1^), R848, or the combination for 12 days, as described in Figure S4C in the Supporting Information, shown by the proportions of the parent gates. Representative flow cytometric plots of total CD45^+^ immune cells G), neutrophils (CD45^+^CD3^−^NK1.1^−^CD11b^+^Gr1^+^) I), and Tregs (CD45^+^CD3^+^CD4^+^CD25^+^Foxp3^+^) K) in the 4 treatment groups are shown. The corresponding proportions of immune cells H), neutrophils J), and Tregs L) after the indicated treatments were quantified by flow cytometry (*n* = 5 or 6 mice per group). The main flow cytometry gating scheme is shown in Figures S4C and S7D in the Supporting Information. M) Representative image of macrophages (CD11b^+^F4/80^+^CD11c^−^DAPI^+^) identified by multiplex immunofluorescence staining in the 4 treatment groups. N) The percentage of macrophages in (M) is shown in a bar graph (*n* = 3 mice per group). Scale bar, 100 µm. The error bars indicate the means ± SEMs; * *p* < 0.05, ** *p* < 0.01, *** *p* < 0.001, **** *p* < 0.0001, Fisher's exact test B–F), one‐way ANOVA H–N).

Therefore, we next determined the immune cell fractions in tumor samples from each treatment arm of an experiment performed with the Hepa1‐6 mouse model using single‐cell RNA sequencing (scRNA‐seq), flow cytometry, and multiplex immunofluorescence staining. The detailed cellular landscape showed differences in the immune composition and epithelial tumor cells among the four treatment groups, partitioning these cells into 11 clusters via scRNA‐seq analysis based on specific identified marker genes (**Figure** **3**A; and Figure S4A,B, Supporting Information). In particular, a significant reduction in the number of tumor cells and an increase in the number of immune cells were observed in the R848 and combination groups compared to those in the vehicle group (Figure 3B,C). Only combination treatment increased the proportion of macrophages (Figure 3D), and multiplex immunofluorescence staining for macrophages (CD11b^+^F4/80^+^CD11c^−^) further validated this result (Figure 3M,N). Both R848 alone and the combination therapy improved neutrophil infiltration and reduced the Treg population (Figure 3E,F). Flow cytometry results further verified that the infiltration of CD45^+^ immune cells was significantly increased (Figure 3G,H), the proportion of neutrophils was increased (Figure 3I,J), and the density of Tregs was decreased after combination therapy (Figure 3K,L). These results ultimately indicated remodeling of the tumor immune microenvironment and inhibition of the immunosuppressive component in the combination group.

### 2.3 The Combination of R848 and Low‐Dose Sorafenib Increases the Proportions and Activation of Antitumor Macrophages and Neutrophils

**Figure 4:**
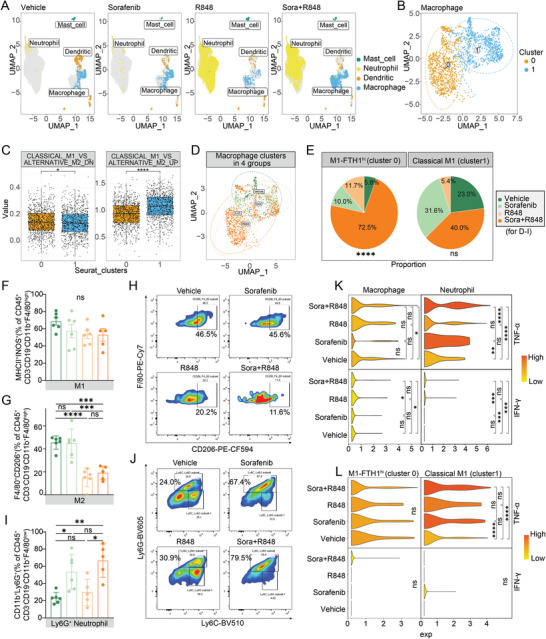
Combination treatment with R848 and low‐dose sorafenib increases the proportion and activation of antitumor macrophages and neutrophils. A) UMAP plots of tumor tissues showing myeloid cells, including mast cells, neutrophils, DCs, and macrophages. Each cell is shown in a different color based on the analysis of single‐cell transcriptome data. B) Unsupervised UMAP clustering identified two macrophage clusters (cluster 0 and cluster 1) in tumor tissues after the combination treatment. C) GSEA of the above mentioned two macrophage clusters based on the gene expression profile enrichment score linked the signals of genes downregulated or upregulated in classical M1 macrophages versus alternative M2 macrophages using GSE5099. D) The distribution of these two macrophage clusters in the 4 treatment groups. E) Pie chart showing the proportions of these two macrophage types (FTH1^high^ M1 (left) and classical M1 (right)) in tumor tissues after vehicle, sorafenib (10 mg kg^−1^), R848, and combination treatment. F–J) The proportions of M1 macrophages (CD45^+^CD3^−^CD19^−^CD11b^+^F4/80^high^MHCII^+^INOS^+^) F), M2 macrophages (CD45^+^CD3^−^CD19^−^CD11b^+^F4/80^high^CD206^+^) G) and Ly6G^+^ neutrophils (CD45^+^CD3^−^CD19^−^CD11b^+^F4/80^low^Ly6G^+^) I) after the indicated treatments were quantified by flow cytometry (*n* = 5 or 6 mice per group). Representative flow cytometric plots of M2 macrophages H) and Ly6G^+^ neutrophils J) in the 4 treatment groups. K,L) Expression levels of TNF‐*α* and IFN‐*γ* in different types of macrophages and neutrophils after the indicated treatments. The error bars indicate the means ± SEMs; ns: *p* > 0.05, * *p* < 0.05, ** *p* < 0.01, *** *p* < 0.001, **** *p* < 0.0001, one‐way ANOVA E,F,G,I), *t*‐test C,K,L).

**Figure 5:**
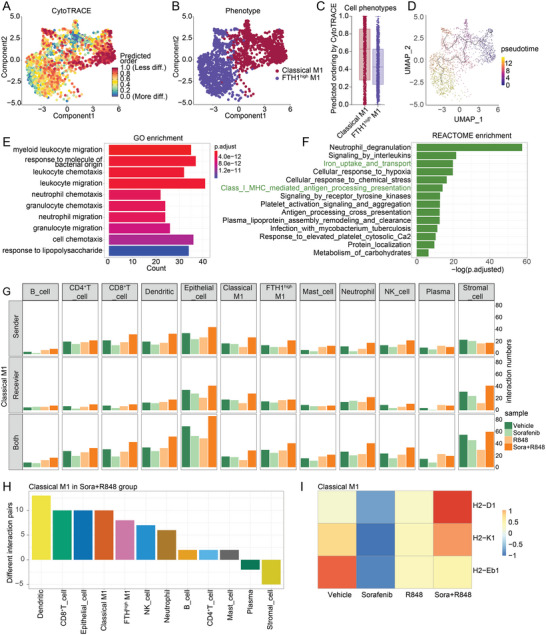
Combination treatment with R848 and low‐dose sorafenib promotes the transition of classical M1 macrophages into FTH1^high^ M1 macrophages and enhances the DC–classical M1 interaction, resulting in enhanced tumor antigen presentation to T cells. A,B) CytoTRACE prediction of the differentiation states in the two macrophage phenotypes based on scRNA‐seq data. The predicted order value indicates the degree of differentiation. C) Boxplot showing the comparison of the predicted ordering between classical M1 and FTH1^high^ M1 macrophage subsets by CytoTRACE. D) Combined application of CytoTRACE and Monocle3 to predict the origin of FTH1^high^ M1 macrophages and verify the trajectories from classical M1 to FTH1^high^ M1 macrophages along pseudotime. E) Gene ontology (GO) enrichment analysis and F) Reactome enrichment analysis of the upregulated genes during transition of M1 macrophages into FTH1^high^ M1 macrophages. *P* values were adjusted for multiple test correction using the Benjamini–Hochberg procedure, and differences were considered significant when adjusted *p* values were less than 0.05. G) CellPhoneDB analysis showed the interactions between classical M1 macrophages and other cells in the TME based on ligand–receptor interactions in the 4 treatment groups. H) The bar graph shows the numbers of ligand–receptor interaction pairs between classical M1 macrophages and other cell types observed in the combination group. I) Heatmap showing the levels of H2‐D1, H2‐K1, and H2‐Eb1 in classical M1 macrophages in the 4 treatment groups. Red color represents an expression level above mean and blue color represents expression level lower than mean.

TLRs act as key mediators of innate immunity, and the TLR7/8 agonist (R848), alone or in combination with sorafenib, altered the proportion of macrophages and neutrophils observed in the myeloid immune cell landscape (**Figure** **4**A). Notably, macrophages and neutrophils are highly diverse and plastic, with a continuum of phenotypes and different activation statuses in response to environmental signals.^[^ ^33^, ^34^ ^]^ M1 (classically activated) and M2 (alternatively activated) macrophages represent two extreme phenotypes of macrophages, indicative of antitumor/inflammatory and protumor functions, respectively.^[^ ^34^ ^]^ Two clusters of macrophages (clusters 0 and 1) were identified in tumor tissues from the four groups of C57BL/6 mice through graph‐based clustering via principal component analysis to generate a unified UMAP embedding space (Figure 4B). We then selected the M1 and M2 macrophage datasets in GSE5099 for verification and annotated the screened clusters based on the gene expression profile enrichment score.^[^ ^35^ ^]^ Cluster 1 was more likely to comprise M1 macrophages according to its higher value for classical M1 macrophage‐ versus alternative M2 macrophage‐upregulated gene signals (Figure 4C). Meanwhile, cluster 2, which had a similar gene expression profile to that of M1 macrophages, including extremely high ferritin heavy polypeptide 1 (FTH1) expression, was annotated as FTH1^high^ M1 macrophages (Figure S5A, Supporting Information). Evidence indicates that FTH1 is a marker gene of M1 macrophages and is involved in macrophage polarization by regulating iron metabolism; moreover, its expression is higher in M1 macrophages than in M2 macrophages.^[^ ^36^, ^37^ ^]^ The differences in macrophage cluster distribution among the four groups further showed that the combination treatment slightly increased the proportion of classical M1 macrophages; however, there were no significant differences among the four groups. Specifically, the proportion of FTH1^high^ M1 macrophages was significantly elevated in the combination group, but these were rare in the control and monotherapy groups (combination 72.5% vs vehicle 5.8%, sorafenib 10.0%, and R848 11.7%; *p* < 0.0001) (Figure 4D,E). Additionally, flow cytometric analysis revealed no significant difference in the percentage of M1 macrophages, but a remarkable reduction in the percentage of M2 macrophages, after R848 or combination treatment compared to those after vehicle or sorafenib treatment (Figure 4F–H; and Figure S5B, Supporting Information). Neutrophil subtype determination revealed that combination treatment primarily increased the population of Ly6G^+^ neutrophils (Figure 5I,J). Overall, the combination treatment dramatically inhibited the polarization of macrophages toward the M2 phenotype, skewing them toward M1 macrophages (classical M1 and FTH1^high^ M1 macrophages) and increasing the proportion of Ly6G^+^ neutrophils.

We also evaluated the activation status of macrophages and neutrophils post‐treatment using scRNA‐seq analysis and qPCR. The combination treatment increased the levels of TNF‐*α* and IFN‐*γ* (Figure 4K,L), which contributed to the activation of M1 macrophages^[^ ^38^, ^39^ ^]^ and drove neutrophils toward an antitumor state.^[^ ^33^, ^40^ ^]^ As neutrophils are plastic, there is evidence that they play a dual role in tumor development.^[^ ^33^ ^]^ Antitumor neutrophils can improve immunotherapeutic efficacy by converting a “cold” tumor immune state into a “hot” state in HCC.^[^ ^41^ ^]^ mRNA expression analysis via RT‐PCR showed high expression of M1 macrophage‐related immune‐activating factors, including *IL‐6*, *IL‐12p35*, *TNF‐α*, and *IL‐1β*, and low expression of M2 macrophage‐related immunosuppressive factors, including *IL‐10*, *Rentla*, and *CCL22*, in the combination group compared to those in the vehicle group (Figure S5C, Supporting Information). Overall, the combination of R848 and low‐dose sorafenib reprogrammed the HCC microenvironment into an immunostimulatory state by enhancing the proportions and activation of antitumor macrophages and neutrophils.

### 2.4 The Combination of R848 and Low‐Dose Sorafenib Promotes the Transition of Classical M1 Macrophages to FTH1^high^ M1 Macrophages and Enhances the DC–Classical M1 Interactions, Increasing Tumor Antigen Presentation to T Cells

To further elucidate the relationships among different macrophage clusters in the tumor environment and their antitumor immunity‐related mechanisms after combination therapy, we used CytoTRACE to predict the differentiation states of these clusters from scRNA‐seq data. CytoTRACE is a powerful computational tool that outperforms previous methods and can establish crucial RNA‐based features of developmental potential and identify cellular hierarchies.^[^ ^42^ ^]^ Intratumoral macrophages exhibited different cellular hierarchical differentiation statuses according to the predicted ordering score from the evaluated scRNA‐seq‐based features (**Figure** **5**A). Furthermore, a difference in the differentiation status was observed between the two macrophage clusters; specifically, classical M1 macrophages were poorly differentiated, whereas FTH1^high^ M1 macrophages were well differentiated (Figure 5B,C). Next, we explored these trajectories using Monocle to order cells along a pseudotime gradient, which indicated that FTH1^high^ M1 macrophages were derived from classical M1 macrophages (Figure 5D). Genes for which expression was upregulated in M1 macrophages transitioning into FTH1^high^ M1 macrophages were primarily enriched in signaling pathways related to neutrophil migration and chemotaxis (Figure 5E), which are prerequisites for any subsequent function executed by neutrophils.^[^ ^43^ ^]^ In addition, neutrophil degranulation was found to be enriched through Reactome pathway analysis (Figure 5F). Neutrophils act as the first line of defense; once they reach a site of infection, they can directly eliminate invading pathogens through diverse mechanisms, including degranulation.^[^ ^43^ ^]^ Accordingly, combination therapy induced a transition from the classical M1 phenotype to the FTH1^high^ M1 state in macrophages, in which the associated gene signals facilitated neutrophil migration and recruitment to the TME and promoted neutrophil degranulation to kill tumor cells (Figure 5E,F). In addition to the previously described role, iron uptake and transport and MHC class I‐mediated antigen processing and presentation signaling were also found to be worthy of subsequent attention (Figure 5F). Iron is essential for maintaining rapid cell proliferation and DNA synthesis in various types of tumors.^[^ ^44^ ^]^ It was reported that M1 macrophages can enhance iron uptake and inhibit iron release, resulting in antitumor effects because abnormal tumor growth requires a relatively large amount of iron to meet nutritional needs.^[^ ^37^ ^]^ Therefore, consistent with the transition from the M1 state to the FTH1^high^ M1 state, the significant increase in the proportion of FTH1^high^ M1 macrophages (Figure 4E) in the combination group suggests that the combined treatment‐induced M1 macrophage transition to FTH1^high^ M1 macrophages might be involved in iron metabolism, which contributes to antigen processing and presentation and the recruitment of more neutrophils.

Whereas the other cluster of macrophages existed in the combination group, classical M1 macrophages not only exhibited activation of the functions mentioned previously herein, in Figure 4K,L, but also showed stronger interactions with DCs, CD8^+^ T cells, and tumor cells in the combination group compared to those in the other groups (Figure 5G,H) through an analysis with CellphoneDB. “CellphoneDB” was recently developed as a public repository of ligands, receptors, and their interactions to enable a comprehensive, systematic analysis of cell–cell communication molecules using single‐cell transcriptomic data.^[^ ^45^ ^]^ As a result of empirical shuffling, cell‐type specific ligand–receptor pairs are calculated by considering the expression levels of ligands and receptors.^[^ ^46^, ^47^ ^]^ DCs act as the most important antigen‐presenting cells (APCs) and the main stimulators of T cell functions,^[^ ^48^ ^]^ interacting closely with classical M1 macrophages (Figure 5H). Furthermore, classical M1 macrophages expressed significantly higher levels of histocompatibility 2, D region locus 1 (H2‐D1) and histocompatibility 2, K1, K region (H2‐K1) in the combination group compared to those in the vehicle group (Figure 5I). These are orthologs of human MHC class I, but not histocompatibility 2, class II antigen E beta (H2‐Eb1), which is also orthologous to human MHC class II. These findings suggest that the combination therapy can enhance DC–classical M1 macrophage interactions, increase the presentation of more tumor antigens to T cells, and initiate an antitumor immune response.

### 2.5 Combination Therapy with R848 and Low‐Dose Sorafenib Promotes Neutrophil–DC Interactions to Enhance Antigen Presentation T Cells, Resulting in Cytotoxic CD8^+^ T‐Cell‐Mediated Antitumor Immunity in HCC

**Figure 6:**
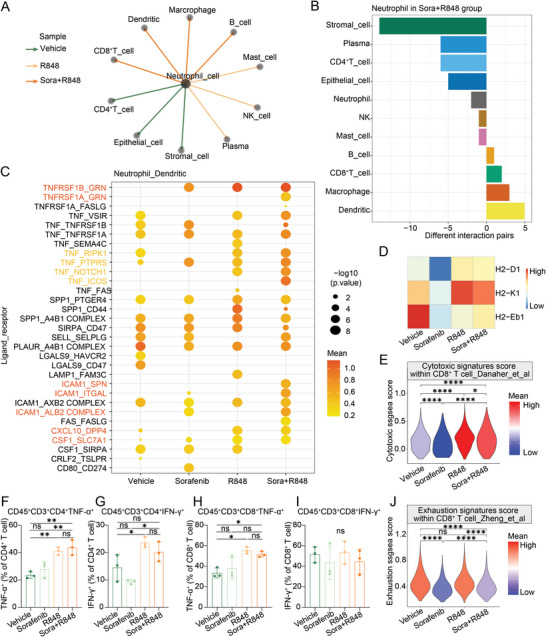
Combination treatment with R848 and low‐dose sorafenib promotes an increase in neutrophil interactions with DCs for antigen presentation to T cells, resulting in CD8^+^ T‐cell‐mediated antitumor immunity. A) Based the CellphoneDB analysis results, each line and its color represent the treatment group in which neutrophils have the most interactions with other cell types among the four groups. B) The bar graph shows the numbers of ligand–receptor interaction pairs between neutrophils and other cell types observed in the combination group. C) Overview of the ligand–receptor interactions between neutrophils and DCs predicted using CellPhoneDB. The statistical significance was estimated using ‐log10 transferred values. D) Heatmap showing the levels of H2‐D1, H2‐K1, and H2‐Eb1 in DCs in the 4 treatment groups. E) ssGSEA of the cytotoxic signature score in CD8^+^ T cells in tumor tissues after the different treatments. F–I) Flow cytometric analysis of the ability to secrete TNF‐*α* and IFN‐*γ* in CD4^+^ T cells and CD8^+^ T cells in the Hepa1‐6 syngeneic mouse model treated with vehicle, sorafenib (10 mg kg^−1^), R848, and combination for 12 days as described in Figure S7 of the Supporting Information, shown by the proportions of parent gates. T‐cell function panel, including the expression of TNF‐*α* and IFN‐*γ* on T cells, shown in bar graph (*n* = 3 per group). J) ssGSEA of the exhaustion signature score in CD8^+^ T cells in the 4 treatment groups. The Error bars indicate the means ± SEMs; ns: *p* > 0.05, * *p* < 0.05, ** *p* < 0.01, *** *p* < 0.001, **** *p* < 0.0001, one‐way ANOVA G–J), *t*‐test F,K).

As shown in Figure 3, in addition to macrophages, neutrophils were the other most commonly increased cell type in the tumor immune microenvironment; therefore, we assessed the communication between neutrophils and other cell types. The CellphoneDB analysis results showed that neutrophils interacted more strongly with DCs and CD8^+^ T cells in the combination group than in the other groups (**Figure** **6**A,B; and Figure S6A,B, Supporting Information). To further identify the key mediators and cell–cell communication mechanisms of neutrophils and DCs, we evaluated putative crosstalk with CellphoneDB based on the expression of ligand–receptor pairs. We found that neutrophils could directly contact DCs through an adhesive ligand–receptor pair, the ICAM1–SPN/ITGAL/ALB2 complex (Figure 6C). In addition, it was found that neutrophils might enhance the chemotactic ability and affinity of DCs via the expression of chemokines and cytokines, including CXCL10^[^ ^49^, ^50^ ^]^ and CSF1 (Figure 6C). Furthermore, neutrophils induced the maturation of DCs via the tumor necrosis factor receptor superfamily members TNFRSF1B/TNFRSF1A‐GRN and TNF‐related signaling molecules TNF‐RIPK1/PTPRS/NOTCH1/ICOS (Figure 6C). These results were consistent with the increase in TNF‐*α* levels in neutrophils shown in Figure 4K, which was previously reported to induce the maturation of antigen‐presenting DCs to trigger robust proliferation of T cells.^[^ ^51^, ^52^ ^]^ The increased expression of H2‐D1 and H2‐K1 in DCs further indicated that combination treatment enhanced the antigen presentation ability of DCs (Figure 6D).

To further identify the effects of myeloid cells on the T cell‐mediated adaptive immune response, we performed single sample gene set enrichment analysis (ssGSEA) of the cytotoxic signature in CD8^+^ T cells and a flow cytometric assay to assess the activity of CD4^+^ and CD8^+^ T cells (Figure 6E; and Figure S7, Supporting Information).^[^ ^53^, ^54^, ^55^, ^56^ ^]^ The cytotoxic signature of CD8^+^ T cells (Figure 6E; and Figure S7A,B, Supporting Information), the ability of CD4^+^ T cells to secrete both TNF‐*α* and IFN‐*γ* (Figure 6F,G; and Figure S7E, Supporting Information), and the ability of CD8^+^ T cells to secrete TNF‐*α*, but not IFN‐*γ*, were significantly enhanced in the combination treatment group (Figure 6H,I; and Figure S7E, Supporting Information). Consistently, a decrease in the exhaustion signature score of CD8^+^ T cells was observed in the combination group compared with that in the vehicle group (Figure 6J; and Figure S7C, Supporting Information). These results indicate that the combination of R848 and low‐dose sorafenib promoted the cytotoxic activity of CD8^+^ T cells and alleviated their exhaustion. Finally, no differences were found in myeloid cells in the blood samples, suggesting that the systemic immune response occurred mainly in the tumor environment (Figure S8, Supporting Information). Overall, the combination of R848 and low‐dose sorafenib promoted increased macrophage–neutrophil interactions with DCs to induce the presentation of antigens to T cells, resulting in a CD8^+^ T cell‐mediated adaptive immune response against the tumor.

### 2.6 Low‐Dose Sorafenib Alone or in Combination with R848 Normalizes the Tumor Vasculature

**Figure 7:**
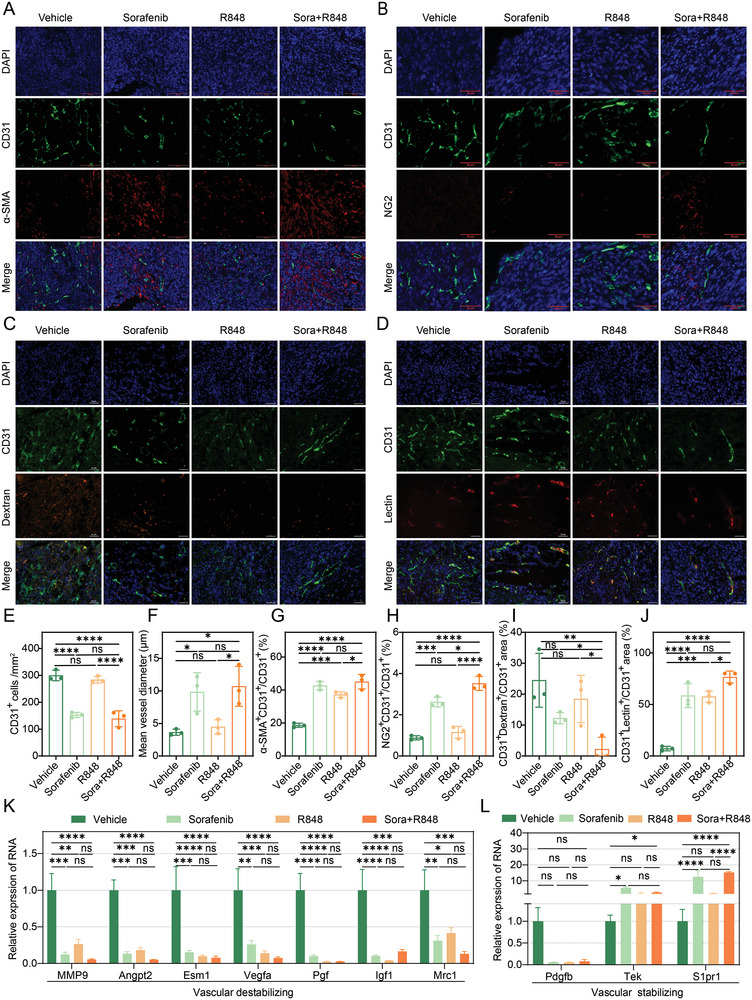
Treatment with low‐dose sorafenib alone or in combination with R848 normalizes the tumor vasculature. A–D) Representative immunofluorescence images of CD31 (green), *α*‐SMA, NG2, dextran and lectin (red), and DAPI (blue) staining in tumor tissues from Hepa1‐6 syngeneic mouse models treated with vehicle, sorafenib (10 mg kg^−1^), R848 or sorafenib+R848 for 12 days. Scale bars, 100 µm A), 50 µm B–D). E–J) Immunofluorescence image quantification results. Relative number of CD31^+^ cells E), tumor vessel diameter F), relative proportions of *α*‐SMA^+^ covered blood vessels G), NG2^+^ pericyte‐covered blood vessels H), dextran^+^ blood vessels I), and lectin^+^ blood vessels J) in tumor tissues from Hepa1‐6 syngeneic mouse models treated with vehicle, sorafenib (10 mg kg^−1^), R848 or sorafenib+R848 for 12 days. K,L) The expression levels of genes associated with vascular distability K) or stability L) detected by RT‐PCR in tumor tissues from mice treated with vehicle, sorafenib (10 mg kg^−1^), R848 or sorafenib+R848 for 12 days. The error bars indicate the means ± SEMs; ns: *p* > 0.05, * *p* < 0.05, ** *p* < 0.01, *** *p* < 0.001, **** *p* < 0.0001, one‐way ANOVA E–L).

Given that immunostimulatory reprogramming and vascular normalization could be mutually regulated,^[^ ^27^ ^]^ studies have revealed that although liver cancer is a highly vascularized tumor, the excessive inhibition of VEGF mediated by high doses of anti‐angiogenic drugs, including sorafenib, can induce an immunosuppressive microenvironment.^[^ ^57^, ^58^ ^]^ To further explore whether the combination of low‐dose sorafenib and R848 evaluated in this study could normalize the vasculature and facilitate the establishment of an antitumor immune microenvironment, we examined the effects of treatment on vascular structure and function. We found that low‐dose sorafenib and combination treatment reduced the total vessel number (**Figure** **7**A–E) but increased the mean vessel diameter (Figure 7F), indicating that sorafenib significantly impaired tumor angiogenesis. Pericytes and vascular smooth muscle cells are required to support the endothelial layer of blood vessels and to maintain vessel maturation and stability.^[^ ^59^, ^60^ ^]^ Upon low‐dose sorafenib or combination treatment, pericyte coverage and vascular smooth muscle cells were significantly increased, as visualized by costaining of neural/glial antigen 2 (NG2) or *α*‐smooth muscle actin (*α*‐SMA), respectively, with CD31 (Figure 7A,B,G,H).

Next, we evaluated whether the structural changes in the tumor vasculature that occurred in response to low‐dose sorafenib or combination treatment could translate into functional improvement. Vascular leakage was significantly attenuated, as detected by monitoring fluorescein‐labeled dextran administered via the tail vein, in the combination group compared to that in the control and R848 groups (Figure 7C,I). Further, vascular perfusion was remarkably promoted, as evaluated by monitoring fluorescein‐labeled lectin after treatment with a low dose of sorafenib or combination therapy (Figure 7J). Moreover, the expression levels of proangiogenic markers that destabilize vessels (*Mmp9*, *Angpt2*, *Esm1*, *Vegfa*, *Pgf*, *Igf1*, and *Mrc1*) were significantly downregulated (Figure 7K), whereas those of vessel maturation‐related markers (*Tek* and *S1pr1*) were upregulated after combination treatment compared to levels with the vehicle treatment (Figure 7L). Consistent with the RT‐PCR results, western blot detection indicated that a vascular‐stabilizing signature in tumors after the combination treatment of low‐dose sorafenib and R848 (Figure S9, Supporting Information). These results, combined with previous findings showing increased IFN‐*γ* secretion by T cells, are consistent with other studies that have reported that IFN‐*γ* can increase the expression of adhesion molecules, such as ICAM1, and decrease endothelial VEGFA expression, which in turn promotes the infiltration of immune cells.^[^ ^27^ ^]^ In summary, low‐dose sorafenib, alone or in combination with R848, normalizes the tumor vasculature, which is associated with a probable positive feedback loop to establish an antitumor immune environment.

## 3 Discussion

**Figure 8:**
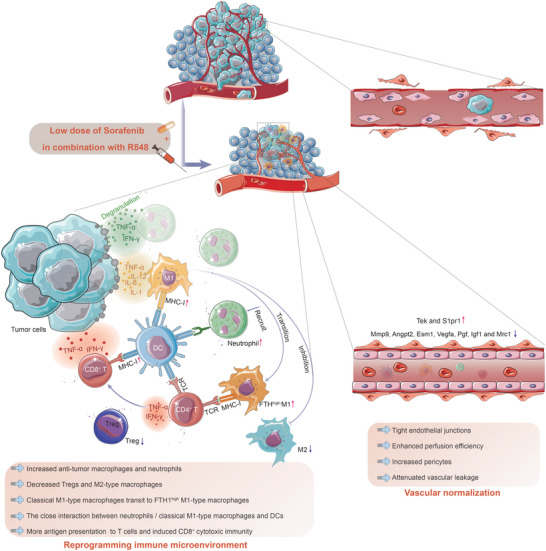
Schematic of the mechanism of interaction between low‐dose sorafenib and R848 in the syngeneic mouse model of HCC. Combined treatment with low‐dose sorafenib and R848 reprograms the tumor immune microenvironment by increasing the population and activation of antitumor macrophages and neutrophils and inhibiting immunosuppressive signaling. The transition of classical M1 macrophages to FTH1^high^ M1 macrophages facilitates an increase in neutrophil recruitment, an increase in iron uptake, and inhibition of iron release for tumor growth tropism after combination treatment. Then, the close interaction of neutrophils/classical M1 macrophages with DCs via some ligand–receptor pairs enhances antigen presentation by DCs through MHC class I molecules, allowing enhanced tumor antigen presentation to cytotoxic CD8^+^ T cells, resulting in antitumor immunity in HCC. TNF‐*α* secreted by T cells contributes to vascular normalization, and the vascular‐normalized environment induced by the combination treatment in turn supports immune cell infiltration and drug perfusion, forming an antitumor positive feedback loop.

Treatment options for HCC remain limited because of the aggressive nature of this cancer. Sorafenib has been widely used as a first‐line drug for treating advanced HCC for decades. However, it is important to note that only 20% of patients with HCC benefit from the use of sorafenib owing to toxicity, acquired resistance, tumor heterogeneity, and immunosuppressive environments.^[^ ^61^, ^62^ ^]^ Likewise, although immune checkpoint inhibitors have achieved unprecedented success for the treatment of tumors in recent years, only a subset of patients respond to these agents. Additionally, the serious adverse reactions and failures of phase III clinical trials combining antiangiogenic agents and PD‐1 antibodies highlight the need to explore new combinations to enhance the clinical benefits of sorafenib^[^ ^9^, ^13^, ^14^ ^]^ (NCT03713593). R848 has been used as an agonist of TLR7/8 in a variety of tumors and as an immune adjuvant to generate antitumor immune memory.^[^ ^63^, ^64^ ^]^ Furthermore, TLR7/8 agonists, rather than TLR3 and TLR9 agonists, can eradicate tumors that evade immune surveillance upon MHC class I downregulation.^[^ ^65^ ^]^ In this study, we directly addressed three critical issues in HCC using a clinically relevant mouse model. First, we developed a novel combination therapy and demonstrated that the antiangiogenic agent sorafenib could be successfully combined with the immunostimulatory agent R848 to increase its therapeutic efficacy in HCC. Second, we showed that lowering the dose of sorafenib could drastically reduce its adverse effects and overcome the toxicity issues. Third, we elucidated an unexpected mechanism underlying the benefits of combination therapy in HCC. Here, we showed that sorafenib/R848 combination therapy did not cause the pruning of abnormal vessels, but instead promoted normalized vessel formation, contributing to immune cell infiltration and drug delivery. Finally, the interactions between normalized vessels and immune cells achieved significant tumor suppression by reprogramming the immune microenvironment of HCC (**Figure** **8**).

To identify available combinatorial cancer treatments, antiangiogenic agents and multikinase inhibitors are among the most notable candidates because of their immunomodulatory capacities.^[^ ^66^, ^67^ ^]^ VEGF has been identified as a key “hub” that reprograms the TME into an immunosuppressive landscape.^[^ ^68^ ^]^ However, whereas sorafenib is a multikinase inhibitor that targets RAF/MEK/ERK signaling and an antiangiogenic agent that targets the VEGFR2, VEGFR3, and PDGFR‐*β* families, it remains unknown whether titrating the dose of sorafenib can induce changes in the TME in HCC, which will lead to benefits upon combinations with immunostimulatory therapy. Our study is the first to report that a lower dose of sorafenib could promote antitumor activity when combined with R848, which was characterized based on its associated good safety, improved ORR, prolonged OS, and even probable immune memory, preventing recurrence. The proposal of a combination strategy is critical and timely for this disease, for which several multitargeted TKIs, including sorafenib, lenvatinib, and regorafenib, have been approved by the FDA as monotherapies. As an added benefit, reducing the dose of a TKI might also mitigate concerns about toxicity in HCC patients, who often have impaired liver functions.^[^ ^69^ ^]^

Increasing studies suggest that low doses of anti‐VEGFA or anti‐VEGFR2 drugs can normalize vascular function, whereas high doses can aggravate hypoxia and accelerate cancer metastasis.^[^ ^70^, ^71^, ^72^, ^73^ ^]^ Gene signals related to vessel normalization are closely associated with immune activation pathways.^[^ ^74^, ^75^ ^]^ Some studies have shown that vascular normalization can improve intratumoral T cell infiltration and the polarization of macrophages into antitumor M1 macrophages^[^ ^67^, ^76^, ^77^ ^]^ through vessel maturation and the relief of immunosuppression induced by hypoxia and/or VEGF.^[^ ^66^, ^78^ ^]^ In turn, CD4^+^ T cells in the tumor environment can induce vascular normalization in an IFN‐*γ*‐dependent manner and further increase immune cell infiltration.^[^ ^74^, ^75^, ^79^ ^]^ Results based on our syngeneic HCC mouse model showed similar results. We found that after treatment with low‐dose sorafenib in combination with R848, tumor vascular normalization occurred, the infiltration of immune cells, including antitumor macrophages and neutrophils, increased, and the polarization of tumor‐associated macrophages into the M2 phenotype was inhibited. Although sorafenib alone could also induce some degree of vascular normalization, it was not sufficient to initiate antitumor immune activity, further emphasizing the important role of R848 as an immune activator based on the normalized vascular microenvironment. Given the complexity and intricate mechanisms of the interaction between vessel normalization and antitumor immunity, the increased secretion of IFN‐*γ* by CD4^+^ T cells induced by combination therapy could be one of the mechanisms linking the mutual regulatory loop between vascular normalization and the immune response. Although more mechanisms need to be explored, this evidence indicates that the combination of immunotherapy and tumor vasculature normalization, rather than destruction of the tumor vasculature, might be a promising anticancer strategy.

Defects in TAA presentation are one of the major factors of immune escape and thus drug tolerance in HCC.^[^ ^80^, ^81^ ^]^ TAAs are displayed on the cell surface via MHC class I molecules. To elicit an effective antitumor response, antigen presentation must succeed as a key event; specifically, the cancer antigen must be taken up by professional APCs, primarily DCs, and cross‐presented to CD8^+^ T cells to achieve priming, recognition, and killing. Tumors utilize multiple evasion mechanisms to avoid immune recognition. Hepatocarcinogenesis is accompanied by inflammation and angiogenesis, in which the established HCC microenvironment forms an anti‐inflammatory matrix and recruits immunosuppressive immune cells, such as Tregs and tumor‐associated macrophages, which block the antigen presentation process and directly inhibit the proliferation and cytotoxic function of CD8^+^ T cells.^[^ ^82^, ^83^ ^]^ Our results showed that combination treatment with low‐dose sorafenib and R848 not only suppressed immunosuppressive signaling, including that in Tregs and M2 macrophages, but also enhanced the antigen presentation capacity of DCs through elevated MHC class I expression. Specifically, the close interaction between activated neutrophils/classical M1 macrophages and DCs via ligand–receptor pairs allowed increased tumor antigen presentation to CD8^+^ cytotoxic T cells, resulting in antitumor immunity against HCC. Therefore, our proposed combination therapy might address the antigen presentation deficiency, as MHC class I presentation of tumor antigens is crucial for immunotherapies aimed at stimulating antitumor CD8^+^ T cell responses.

In addition, using single‐cell transcriptome sequencing combined with flow cytometric analysis, we found that the combination treatment promoted the conversion of proinflammatory M1 macrophages into FTH1^high^ M1 macrophages, but not M2 macrophages. Ferritin is an iron storage protein that plays a key role in iron metabolism and affects antitumor immunity.^[^ ^84^ ^]^ H‐ferritin (Fth, also known as FTH1) exerts proinflammatory effects on M1 macrophages by increasing intracellular iron levels.^[^ ^84^, ^85^ ^]^ Iron is an essential component of many proteins involved in cell growth and replication, and tumor cells require more iron than normal cells because they typically proliferate more rapidly.^[^ ^86^ ^]^ Therefore, iron chelators have shown good antitumor activity in cell culture experiments and clinical trials.^[^ ^87^ ^]^ Hence, the two macrophage clusters identified after combination treatment, classical M1 macrophages and FTH1^high^ M1 macrophages, might have different antitumor functions, with one based on classical proinflammatory activity and the other mainly related to iron uptake. These findings provide a better understanding of macrophage subtypes and functions.

Neutrophils are key players in the innate immune system, providing the first line of defense against invading pathogens, and can directly combat pathogens through degranulation and the release of neutrophil extracellular traps. Some studies have shown that neutrophils can interact with DCs, driven by the binding of DC‐specific C‐type lectin (DC‐SIGN) to Mac‐1/CEACAM1. Upon activation, neutrophils release TNF‐*α* to induce DC maturation, enabling these DCs to trigger strong T cell immunity.^[^ ^51^ ^]^ In line with this, we demonstrated that neutrophil–DC interactions, mediated by ligand–receptor pairs, facilitated more tumor antigen presentation to cytotoxic CD8^+^ T cells, resulting in antitumor immunity in HCC. Neutrophils play a dual role in tumor development because of their plasticity, and antitumor neutrophils can improve the immunotherapeutic efficacy by converting a “cold” tumor immune state into a “hot” state in HCC.^[^ ^41^ ^]^ Taken together, our data define a molecular pathway to establish a cellular link between DCs and neutrophils, thus suggesting a novel cellular connection between innate and adaptive immunity in HCC. The increased numbers of neutrophils observed after combination treatment might facilitate the establishment of an immunostimulatory microenvironment.

## 4 Conclusion

In summary, our study provides a new approach for combination treatment. The immune activator R848 combined with subclinical doses of sorafenib effectively increased antitumor activity with little toxicity. Combination treatment reprogrammed the tumor immune microenvironment by inhibiting immunosuppressive signals and increasing the infiltration and activation of antitumor macrophages and neutrophils. In addition, combination treatment enhanced the antigen‐presentation capacity by increasing the interactions of neutrophils and macrophages with DCs to initiate an immune response mediated by CD8^+^ T cells. Our findings provide preclinical evidence that R848 could be a promising therapeutic candidate for HCC, and its combination with sorafenib might warrant further clinical investigation.

## 5 Experimental Section

### Mice and Cell Lines

C57BL/6 mice (males, 4–8 weeks of age) were purchased from Gempharmatech (Jiangsu, China) and housed in the SPF facility of Tianjin Medical University Cancer Institute and Hospital. Mouse liver cancer Hepa1‐6 cells were purchased from the American Type Culture Collection (Manassas, VA). The cells were cultured in complete medium (DMEM) supplemented with 10% fetal bovine serum (PAN‐Seratech) and 1% penicillin‐streptomycin solution (HyClone) at 37 °C in a humidified incubator with 5% CO_2_. The study was approved by the Research Ethics Committee of the Tianjin Medical University Cancer Institute and Hospital (approval no. AE2021001), and the study was conducted in compliance with the Declaration of Helsinki.

### Tumor Therapy

Hepa1‐6 cells (1×10^6^ cells per mouse) were subcutaneously injected into the armpit of C57BL/6 mice, as indicated. After the injection, the mice were monitored, and the tumor sizes were measured daily using a caliper. On day 10, the mice were administered medication when the tumor had grown to ≈200 mm^3^. Mice were randomly assigned to four groups as follows: control, sorafenib, R848, and combination groups. Sorafenib (10 mg kg^−1^, 0.2 g dissolved in 5 mL ethanol and 5 mL castor oil) was administered daily via gavage, and R848 (20 µg per mouse, 20 mg dissolved in 1 mL ethanol) was administered twice per week via intratumor injection. Mice in the control group were treated with corresponding solvent (vehicle) by the same route.

### Statistical Analyses

All data were obtained from at least three independent experiments and shown as the means ± SEMs unless otherwise mentioned. The exact sample size for each experimental group and specific statistical tests used to assess significant differences are shown in every Figure as the number of dots or indicated in Figure legends. Two‐tailed unpaired Student's *t*‐test was used to compare the differences between two different treatment groups, whereas analysis of experiments with more than two groups was performed using one‐way ANOVA with Scheffe's correction for multiple comparisons. Survival and time to response were evaluated by constructing Kaplan–Meier curves and using the log‐rank (Mantel–Cox) test. Correlations for categorical variables were analyzed by Fisher's exact test. The statistical analyses were performed using R (version 4.2.1) and GraphPad Prism 7 software. Statistical significance was defined as *p* values less than 0.05: *p < 0.05, **p < 0.01, ***p < 0.001, and ****p < 0.0001 and n.s. indicates no significant difference.

### Ethical Statement

The study was approved by the Research Ethics Committee of Tianjin Medical University Cancer Institute and Hospital and was conducted in compliance with the Declaration of Helsinki.

## Conflict of Interest

The authors declare no conflict of interest.

## Author Contributions

Y.H., Ph.D. (Conceptualization: Lead; Data curation: Lead; Formal analysis: Lead; Funding acquisition: Equal; Methodology: Lead; Writing‐original draft: Lead; Writing‐review & editing: Equal) L.Z., Masters and J.S., Ph.D. (Data curation: Equal; Visualization: Equal; Writing‐original draft: Supporting;) M.X., Masters, R.Z., Ph.D. and C.W., Ph.D. (Data analysis: Supporting; Experiment performation: Supporting) Z.L., Professor (Resources: Supporting) W.G., Ph.D. (Visualization: Supporting) Y.L., Masters (Visualization: Supporting) L.C., Masters (Visualization: Supporting) S.Z. and Y.W., Professor (Conceptualization: Equal; Resources: Equal; Supervision: Equal) C.L., Professor (Conceptualization: Equal; Resources: Equal; Funding acquisition: Equal; Supervision: Equal) H.G., Professor (Conceptualization: Lead; Funding acquisition: Lead; Supervision: Lead; Writing‐original draft: Equal; Writing‐review & editing: Lead).

## Supporting information

Supporting Information

## Data Availability

The data that support the findings of this study are available from the corresponding author upon reasonable request.
