# Supplementary material for: The Combination of R848 with Sorafenib Enhances Antitumor Effects by Reprogramming the Tumor Immune Microenvironment and Facilitating Vascular Normalization in Hepatocellular Carcinoma
Source: Adv Sci (Weinh). 2023 Apr 21;10(18):2207650. doi: 10.1002/advs.202207650 (PMC10288281; doi:10.1002/advs.202207650)
Supplement: Supplementary file 1 — Supporting Information [file ADVS-10-2207650-s001.pdf]

ADVANCED SCIENCE
Open Access

Supporting Information

for *Adv. Sci.*, DOI 10.1002/advs.202207650

The Combination of R848 with Sorafenib Enhances Antitumor Effects by Reprogramming the Tumor Immune Microenvironment and Facilitating Vascular Normalization in Hepatocellular Carcinoma

Yuchao He, Linlin Zhan, Jian Shi, Manyu Xiao, Ran Zuo, Chengmeng Wang, Zhiyong Liu, Wenchen Gong, Liwei Chen, Yi Luo, Shaojun Zhang, Youwei Wang*, Lu Chen* and Hua Guo**

The combination of R848 with sorafenib enhances antitumor effects by reprogramming the tumor immune microenvironment and facilitating vascular normalization in HCC

Yuchao He, Linlin Zhan, Jian Shi, Manyu Xiao, Ran Zuo, Chengmeng Wang, Zhiyong Liu, Wenchen Gong, Liwei Chen, Yi Luo, Shaojun Zhang*, Youwei Wang*, Lu Chen*, Hua Guo*

Supplementary Materials and Methods

TUNEL system

The tumors after treatment were fixed with formalin and made into paraffin sections. DeadEnd™ Fluorometric TUNEL System (Promega, USA) were used according to the instructions.

H&E staining

The paraffin-embedded mouse tumor sections were sequential washed by Xylene and graded concentrations ethanol. Then the sections were stained with hematoxylin and eosin, dehydrated with graded concentrations ethanol, seal with neutral gum, mounted, dried and observed.

Flow cytometric assay

Tumor tissues were separated and cut into small pieces and digested for 1h at 37℃ incubator using Tumor Dissociation Kit, mouse (MACS, Germany). Spleens and livers were grinded in the Copper mesh. Then the cells were filtered through the 40 and 70 μm cell strainers. Blood samples were taken from the orbital sinus of mice and

loaded into a tube containing anticoagulant. All the red blood cells of above tissues were lysed by ACK lysis buffer on ice for 5 minutes. The 1×10^5 cells were stained in 1.5-mL tubes with antibody for 15 minutes at room temperature (25°C) in the dark. Finally, 400 μL PBS was added to each tube. Samples were analyzed on a FACS Aria flow cytometer (BD) with CellQuest software, and the data were analyzed using FlowJo software. The antibodies are listed as follow: NK1.1, Ly6C, F4/80 (eBioscience, USA); Gr-1, CD11b, CD11c，MHC I, CD8, CD69, CD206, CD45 (Biolegend, USA); CD3 (BD bioscience, Germany).

Toxicity study

For toxicity assessment, mice were weighed before and after treatment (on day 21 for R848 and sorafenib). Blood samples were collected from the orbital sinus using a microhematocrit tube after each treatment and subjected to biochemical analysis for kidney function markers blood urea nitrogen (BUN) and creatinine and liver marker enzymes alanine transaminase (ALT) and aspartate transaminase (AST) to evaluate treatment toxicity by COBAS ® 8000 analyzer series (Roche Diagnostics, Rotkreuz, Switzerland).

RNA extraction, cDNA synthesis, and quantitative real-time PCR

Mouse tissue total RNA was isolated from the digested tumor cells using TRIzol reagent (Ambion, USA). cDNA was synthesized by RNA reverse transcription using a quantitative RT-PCR kit (Takara, Japan). The amplification reaction was performed according to the manufacturer’s instructions (Takara, Japan) using predesigned primers. Primer sequences are listed in Table S1, Supporting Information.

Multiplex immunofluorescence and analysis

Mouse tumor tissues were fixed in paraformaldehyde (PFA), paraffin-embedded 4 μm sections were baked for 1 h at 60 °C, then stained according to protocol of PANO 6-plex IHC kit, cat 10236100100 (Panovue, Beijing, China). Primary antibodies were used in following order: Pannel 1 of Macrophage): 1. CD11c (Santa cruz, 398708, 1:50; PPD650); 2. CD163 (Abcam, 182422, 1:100; PPD480); 3. CD11b (Abcam, 133357, 1:100; PPD520); 4. F4/80 (Proteintech, 28463-1-AP, 1:500; PPD570); 5. CD206 (Abcam, 64693, 1:500; PPD780). Pannel 2 of tumor vessels: 1. CD31 (Abcam, 281583, 1:1000; PPD520); 2. α-SMA (Abcam, 124964, 1:1000; PPD570); 3. NG2 (Abcam, 259324, 1:50; PPD650). Subsequently, sections were stained with DAPI (SIGMA-ALDRICH, D9542). Slides were mounted and scanned by a PanoVIEW VS200 slide scanner (Panovue, Beijing, China) with Olympus 40× lens. 5-10 representative multispectral images were selected as training samples to develop an algorithm using inForm Advanced Image Analysis software (inForm 2.5.0; Akoya Biosciences, USA).

In vivo Vascular Leakage and Perfusion Assay and quantification

FITC-conjugated dextran (D1820, Thermo Fisher Scientific, 100 mg kg^{-1}) was injected intravenously, 3 h later, mice were injected i.v. using Lycopersicon esculentum (tomato) lectin conjugated to Dylight 649 (DL-1178-1, Vector Laboratories, 10 mg kg^{-1}), after 10 mins of circulation, the mice were killed. Tumors were collected and 8 μm frozen sections, then stained with CD31 (Abcam, 281583, 1:1000). Images were processed by using an LSM 880 with Airyscan (Zeiss).

Single-cell RNA sequencing (scRNA-seq)

scRNA-seq generation (Single cell isolation/library construction/sequencing)

Following with 10× Genomics® (Novogene, China) Cell Preparation Guide described, mice were euthanized by cervical dislocation, and the tumor tissues were peeled off and made into a single cell suspension after splitting red blood cells by Tumor Dissociation Kit (Miltenyi Biotec, Germany) and ACK lysis buffer. Cell viability should exceed 80% as determined by Taipan Blue staining. Calculate the appropriate volume of cell suspension so that each sample contains approximately 12,000 cells.

Cell suspensions were loaded into Chromium microfluidic chips with 30 v2 chemistry and barcoded with a 10× Chromium Controller (10× Genomics). Captured cells are lysed and transcripts are barcoded by reverse transcription. The scRNA-seq library was constructed using a Chromium Single Cell 30 v2 reagent kit (10× Genomics) according to the manufacturer's instructions. Each sample was processed independently, without excluding either cell. The constructed libraries were sequenced on the Illumina (HiSeq 2000).

Raw reads were transformed into FASTQ files using Illumina sequencers, then checked by FastQC. Cell Ranger pipeline (v.5.0.1, 10× Genomics (https://support.10xgenomics.com/single-cell-gene-expression/software/downloads/5.0)) was used to perform basic statistics.

Raw data processing, data filtering and cell clustering of scRNA-seq

The 10× Genomics Cell Ranger pipeline was used to demultiplex raw reads, read alignment and generation of the gene-cell matrix (10× Genomics, v5.0.1, GRCh38).

Genes detected in less than 3 cells and cells in which detected transcripts were either fewer than 200 genes or >6000 genes and >50% of transcripts derived from mitochondrial genes were filtered out and excluded from the subsequent analysis. And Doublets were removed by DoubletFinder. Seurat R package (version 4.1.0) were used to normalized and scaled the gene count matrix to identify highly variable genes for unsupervised cell clustering via FindVariableFeatures function with default parameter. Principal component analysis (PCA) was performed on top 2000 highly variable genes. The cells were clustered on the basis of the first 30 PCs using FindNeighbors and FindClusters with a resolution set to 0.5. Finally, we manually annotated the cell types using canonical marker genes. The second round of clustering T cells and macrophage cells was the same as above: starting from normalized and scaled the expression matrix, identifying highly variable genes with FindVariableFeatures method, and clustering with FindNeighbors and FindClusters function.

Cell-cell communications analysis

Mouse genes were mapped to their human genes orthologs before analysis. CellPhoneDB (https://www.cellphonedb.org/) was used to anticipate enriched ligand-receptor interactions between two distinct cell types based on single-cell transcriptomics data.

Briefly, on the basis of literature and public databases, a vetted database of ligand–receptor interactions were constructed. The average receptor expression level and ligand expression level for each pair of cell types were calculated using a random permutation of the cell type labels on all cells. The process was repeated 1000 times to

create a null distribution for each ligand–receptor pair in each pair of cell types. *P* value was calculated by computing the percentage of the means that are equal to or higher than the null distribution for a certain ligand–receptor pair. Only receptors and ligands that were generated by more than 30% of the cells in the specific cluster were accounted for.

To further verify the results of CellPhoneDB analysis, we also applied the CellChat algrithms to infer cell-cell communications between immune cell subclusters. The CellChat package includes a comprehensive database of signaling molecule interactions, which takes into account the known structural composition of receptor-ligand interactions, such as multimeric receptor-ligand complexes, stimulatory and inhibitory membrane-bound coreceptors, as well as soluble agonists and antagonists. The inference of CellChat includes identification of ligand-receptor interactions, calculation of signaling pathway interactions pattern. The CellChat analysis evaluated the differences in ligand-receptor interactions among four treatment groups.

Pathway enrichment analysis and ssGSEA analyses

To identify differential expression genes (DEGs), FindMarkers and FindAllMarkers functions in Seurat were performed. By GO and Reactome pathway enrichment analysis, DEGs related to the differentiation from M1 macrophages to FTH1^{high} macrophages was assessed to find enriched pathways. Gene Ontology (GO) enrichment analysis of target genes was developed by the clusterProfiler R package. Reactome pathway-based analysis was performed using the ReactomePA R package. To evaluate the function of DEGs associated with T cells, we used ssGSEA (run by R package GSVA) to evaluate the activity levels of gene sets associated with T cell cytotoxicity and exhaustion for each sample. Only genes were significant ($p<0.05$) and with an

average logFC higher than log (2) were considered.

Trajectory and pseudotime analysis for Single-cell RNA-seq

The R package CytoTRACE v.0.3.3 was used to predict the differentiation state of cells from the single-cell RNA-seq (scRNA-seq) data. R package Monocle 3 was used to discover the differentiation trajectory of M1 macrophage converting into FTH1^{high} macrophage. Utilizing M1 macrophages as the root state, each cell was given a pseudotime value using the order_cells function. We also applied the CytoTRACE algorithm to predict the differentiation state of macrophage. The identified path was mapped to UMAP projection for visualization.

CIBERSORT deconvolution for surgical tissues bulk RNA-seq profiles

CIBERSORT (Cell-type Identification by Estimating Relative Subsets of RNA Transcripts, http://cibersort.stanford.edu) is a computational tool that can estimate the relative fractions of various cell clusters in the gene expression profiles. We imported 37 RNA-seq dates of HCC tissues, which are classified as nonresponse and response to sorafenib, respectively, into CIBERSORT in order to assess the varying ratios of infiltrating immune cells in various response groups to sorafenib. The average mean of 14 different infiltrating-immune cells has been recognized by CIBERSORT-LM22 and was computed individually in the two groups.

Western blot assay

To prepare the tissue proteins, the tumor tissues were first homogenized in a lysis buffer containing protease and phosphatase inhibitors to prevent protein degradation. The lysates were then centrifuged to remove any debris, and the supernatants were

collected as the protein samples. The protein samples were loaded onto an SDS-PAGE gel and separated by size using an electric field. The separated proteins were then transferred onto PVDF membranes (Roche, Basel, Switzerland). After blocking with 3% albumin from bovine serum (BSA) for 1 h, the membrane was incubated with various primary antibodies overnight at 4°C, followed by incubation with corresponding secondary antibodies at a 1:4000 dilution for 1 h at room temperature. The primary antibodies were: anti-β-actin (1:1000; Santa Cruz, sc-47778), anti-Angpt2 (1:500; Bioss, bs-0677R), anti-Tie2 (1:500; Bioss, bs-23638R), anti-Mmp9 (1:500; Bioss, bs-4593R), anti-Pdgfb (1:500; Bioss, bs-0185R), anti-Esm1 (1:500; Bioss, bs-3615R), anti-Mrc1 (1:1000; Abcam, ab64693), anti-S1pr1 (1:1000; Proteintech, #55133-1-AP), anti-Vegfa (1:1000; Abcam, ab46154), anti-Pgf (1:500, Abcam, ab196666).

Supplementary Figures and figure legends

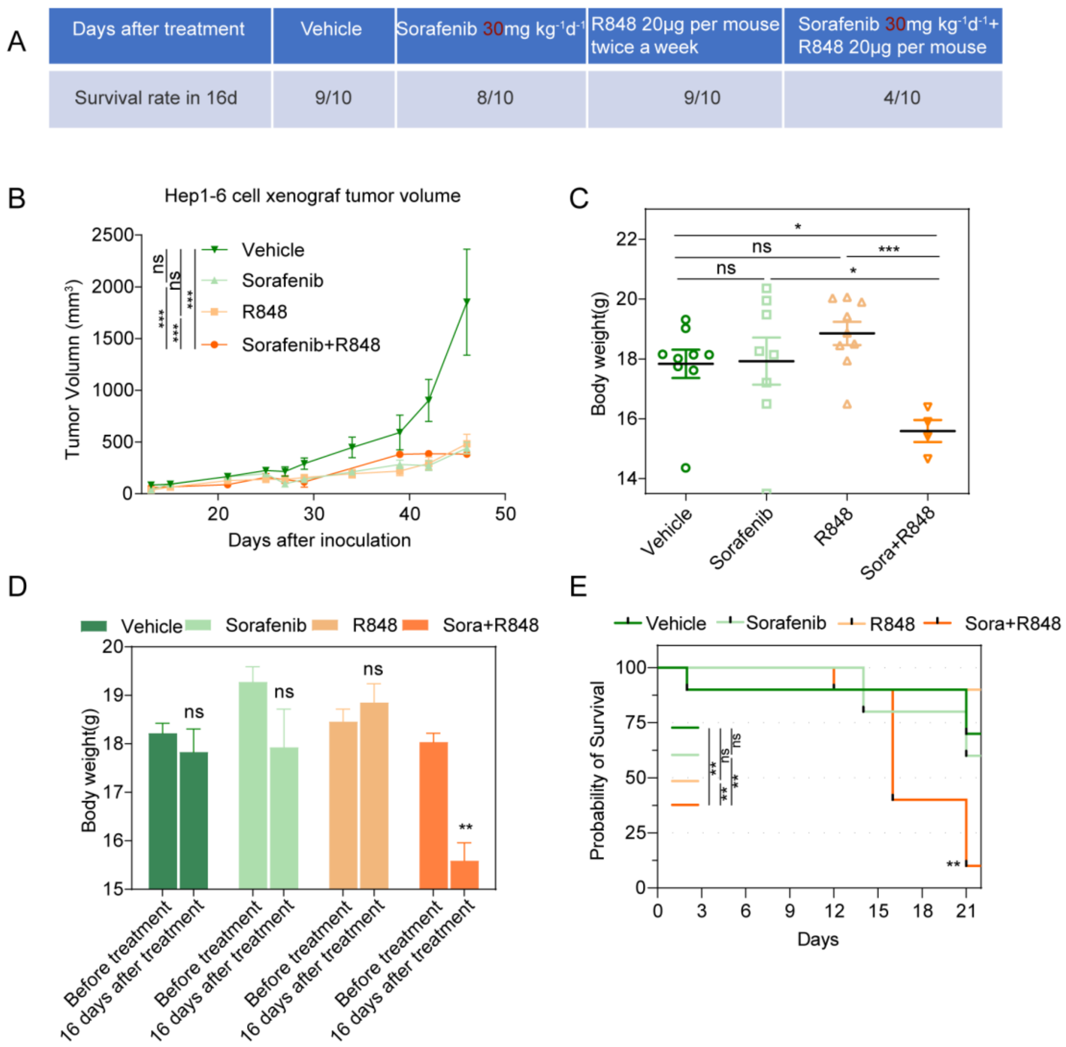

Figure S1. Toxicity induced by high-dose sorafenib (30mg/kg) combination with R848. (A) The survival rates of mice after indicated treatments at 16 days (sorafenib intragastrically (clinical dose, mg kg^{-1}), R848 (20μg per mouse), sorafenib combined with R848, or the vehicle (n=10 per group). **(B)** Tumor growth curve of Hepa1-6 tumors at the indicated time points after treatment of (A). **(C)** The body weight of treated mice at endpoint of (B). **(D)** A comparison of body weight 16 days after treatment to before treatment. **(E)** Kaplan-Meier survival curves of treated mice (n=10 per group). Error bars represent means ± SEMs; ns: $p > 0.05$, * $p < 0.05$, ** $p < 0.01$, *** $p < 0.001$, **** $p < 0.0001$, one-way ANOVA (**B** and **C**), t-test (**D**), log-rank test (**E**).

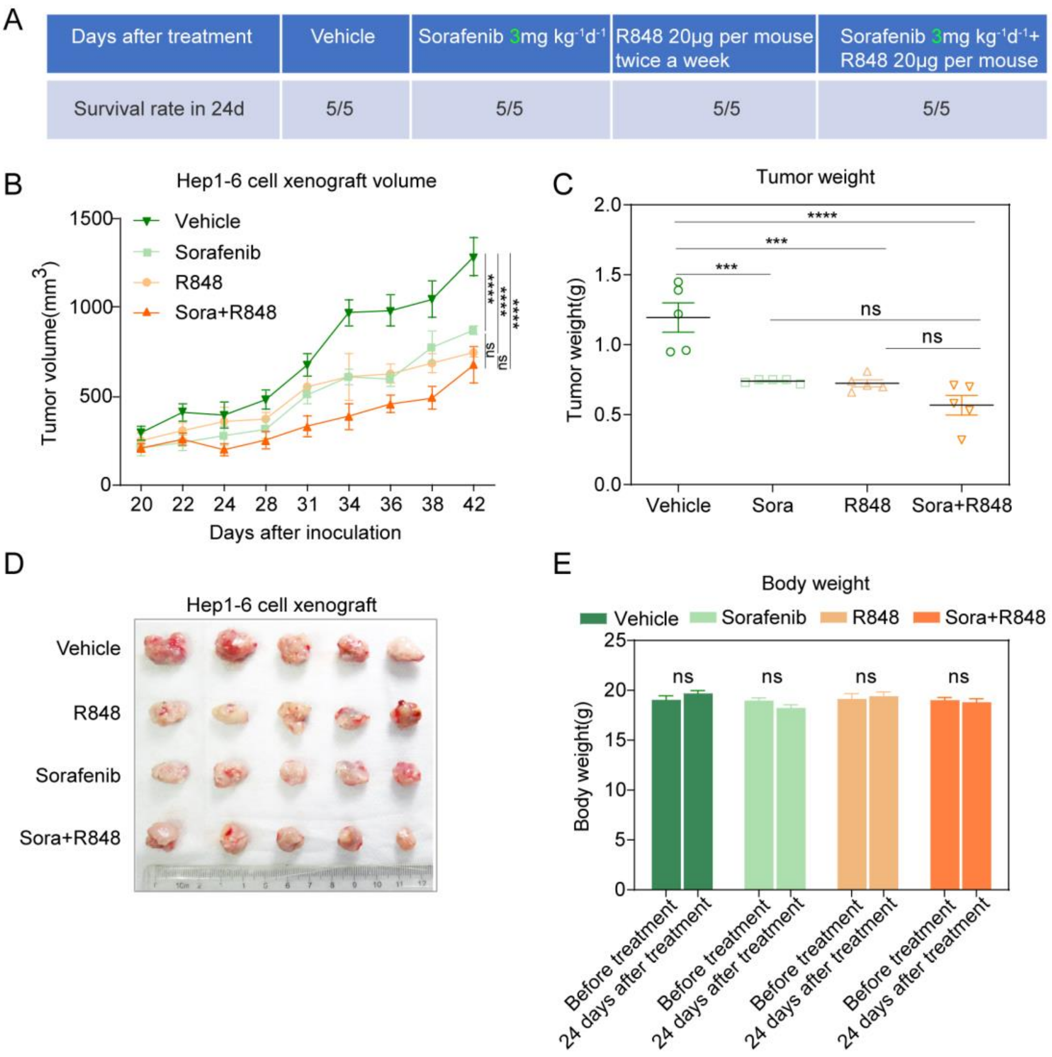

Figure S2. Non-increased efficacy induced by lower dose sorafenib (3mg/kg) combination with R848. (A) The survival rates of mice after indicated treatments at 24 days (sorafenib (3 mg kg^{-1}), R848 (20μg per mouse), sorafenib combined with R848, or the vehicle (n=5 per group). **(B)** Tumor growth curve of Hepa1-6 tumors at the indicated time points after treatment of (A). **(C)** The tumor weight of treated mice at endpoint of (B). **(D)** Representative photographs of Hepa1-6 subcutaneous HCC tumors after indicated treatments of (A). **(E)** A comparison of body weight 24 days after treatment to before treatment. Error bars represent means ± SEMs; ns: $p > 0.05$, * $p < 0.05$, ** $p < 0.01$, *** $p < 0.001$, **** $p < 0.0001$, one-way ANOVA (**B** and **C**), t-test (**E**).

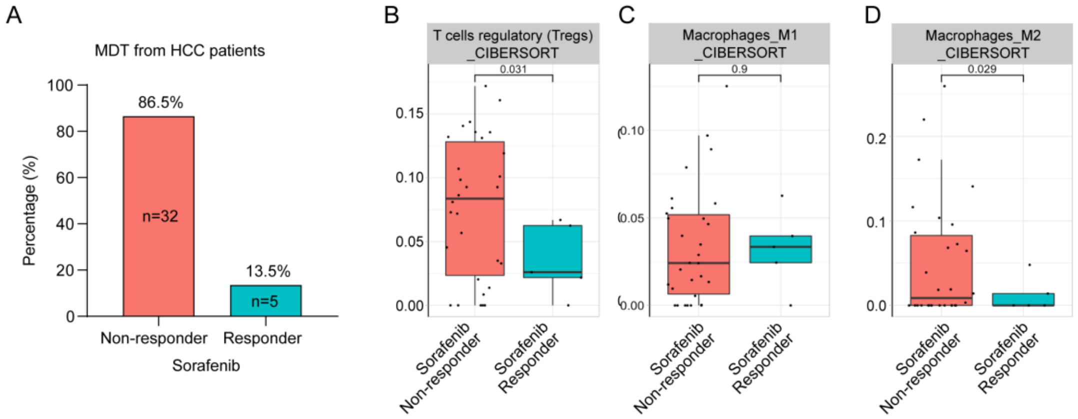

Figure S3. Sorafenib-non-response HCC patients display an immunosuppressive tumor microenvironment. (A) The rates of response to sorafenib in 37 advanced HCC patients by the *ex vivo* method for drug testing, micro-dissected tumor tissues on chip. **(B-D)** The composition of Tregs, M1 and M2 type macrophages in sorafenib non-responder or responder HCC patients based on the bulk tumor tissue gene expression profile analysis. Error bars represent means ± SEMs; ns: $p > 0.05$, * $p < 0.05$, ** $p < 0.01$, ***, $p < 0.001$, ****, $p < 0.0001$, *t*-test (**B-D**).

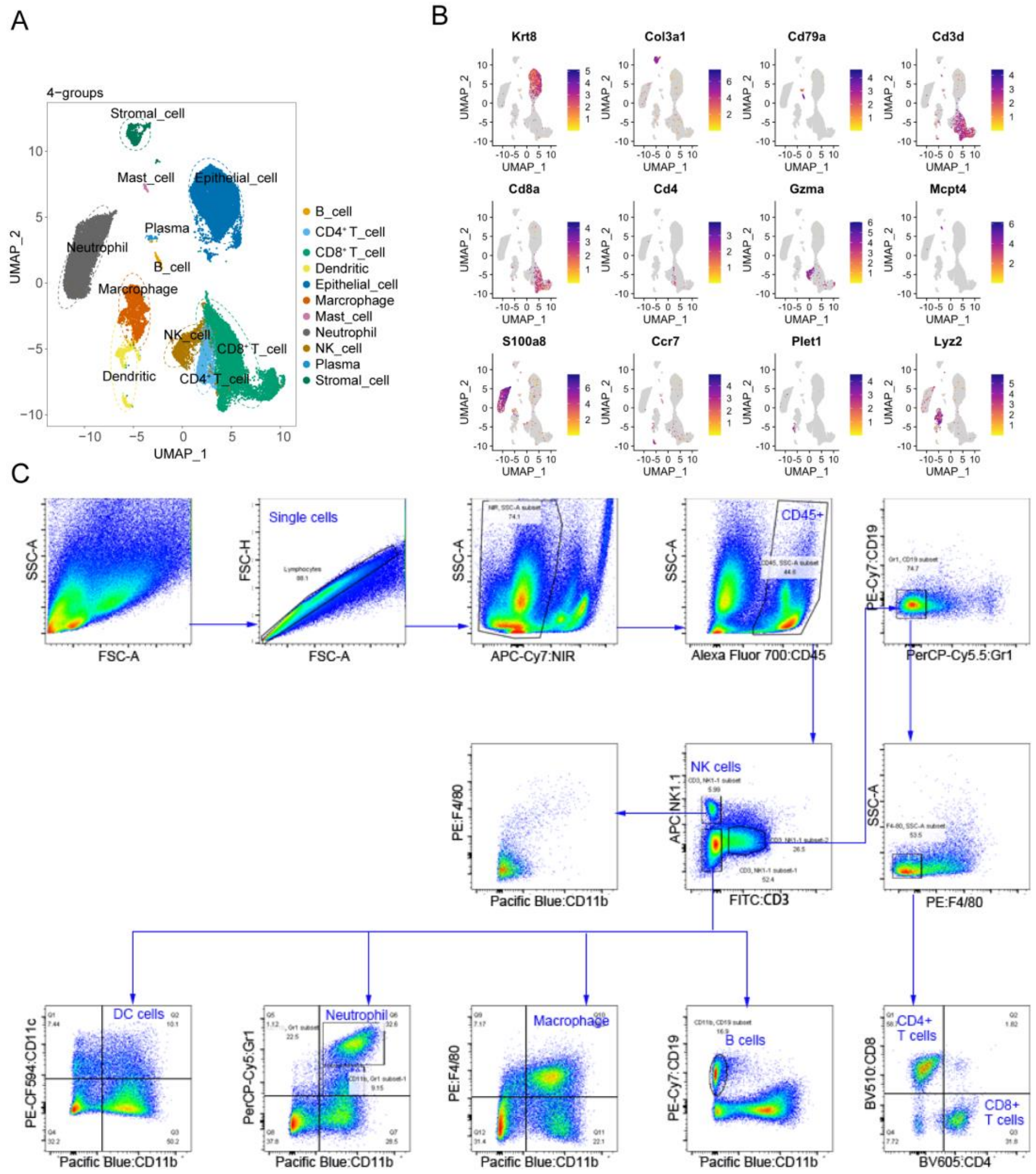

Figure S4. Cell annotation markers in single cell sequencing and work flow of Panel 1 in flow cytometry. (A-B) The UMAP plots of 11 clusters in tumor tissues of single-cell data relies on reference annotations to label cells (**A**), and the expression of respective specific identified marker genes about the 11 cell clusters (**B**). **(C)** Work flow of major immune cell panel in multiparameter flow cytometry analysis.

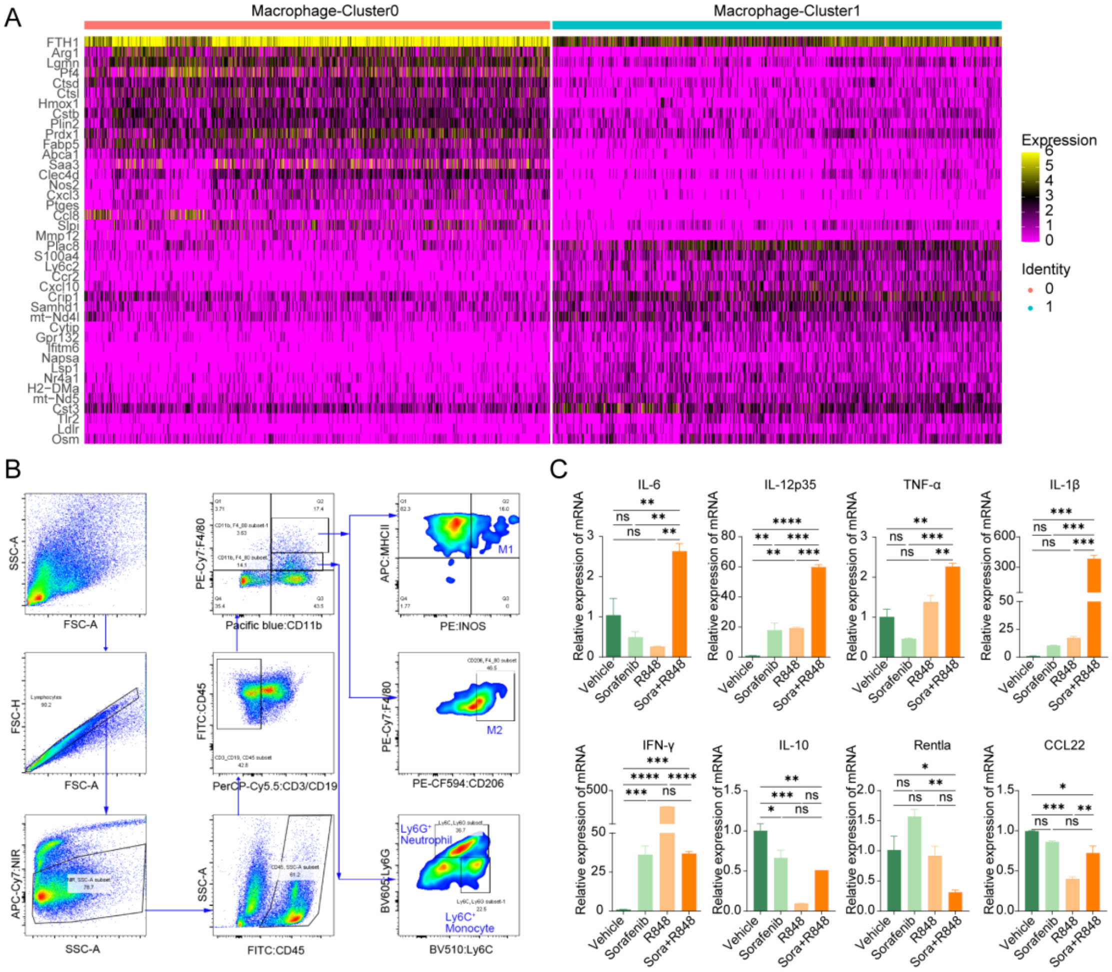

Figure S5. Combination of R848 and low-dose sorafenib promotes the activation of anti-tumor macrophage. (A) Heat map of two macrophage clusters related genes. **(B)** Work flow of macrophage and neutrophil panel in multiparameter flow cytometry analysis. **(C)** The level of macrophages inflammatory factors detected by RT-PCR assay were shown, including M1 macrophages related factors IL-6, IL-12p35, TNF-α, IFN-γ, IL-1β, and M2 macrophages related factors IL-10, Rentla, CCL22. Error bars represent means ± SD; ns: $p > 0.05$, * $p < 0.05$, ** $p < 0.01$, *** $p < 0.001$, **** $p < 0.0001$, two-way ANOVA (**C**).

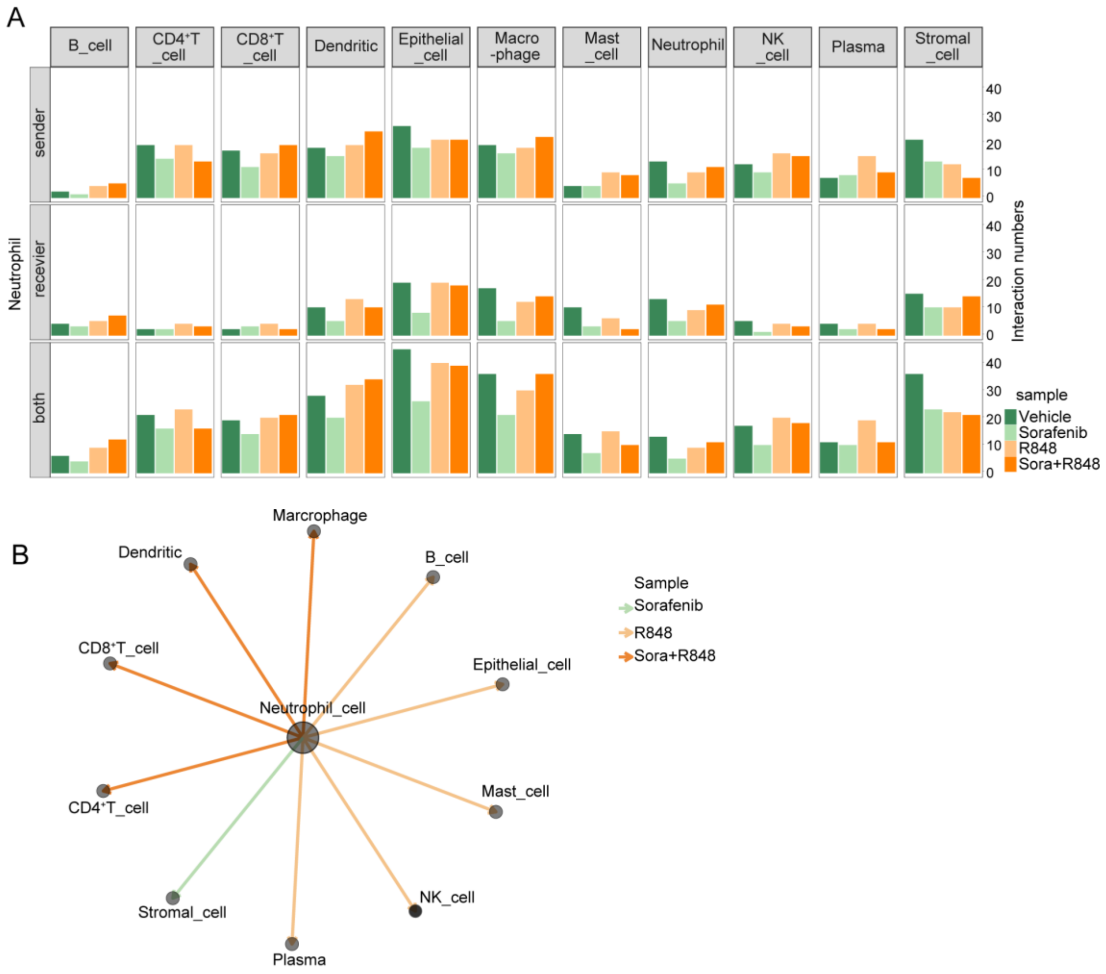

Figure S6. The interactions between neutrophils and other cell types. (A) CellPhoneDB analysis showed the comparison of interactions between neutrophils and other cell types in the TME based on ligand–receptor interactions in the 4 treatment groups. **(B)** The CellChat analysis result revealed the enriched group in which each type of immune cell had the most ligand-receptor pairs when communicating with neutrophils.

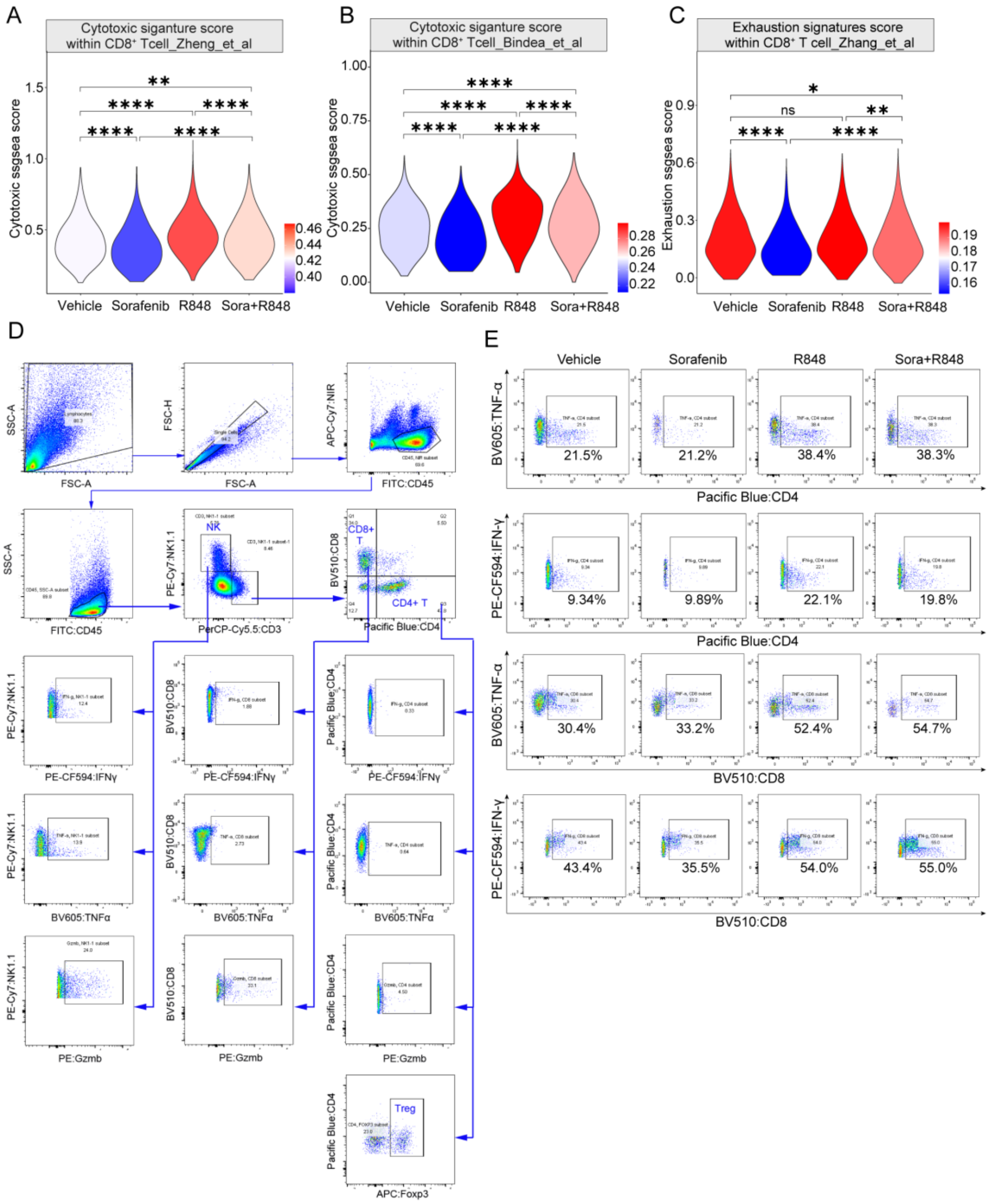

Figure S7. The analysis of T cell function. (A-B) ssGSEA of cytotoxic signature score within CD8$^+$ T cells for each cluster with different treatments. **(C)** ssGSEA of exhaustion signature score within CD8$^+$ T cells in 4 treatment groups. **(D)** Work flow of T cell function panel in multiparameter flow cytometry analysis. **(E)** The representative flow cytometric plot of TNF-α and IFN-γ secreted by CD4$^+$ T cells and CD8$^+$ T cells. ns: $p > 0.05$, * $p < 0.05$, ** $p < 0.01$, *** $p < 0.001$, **** $p < 0.0001$, Fisher's exact test (**A-C**).

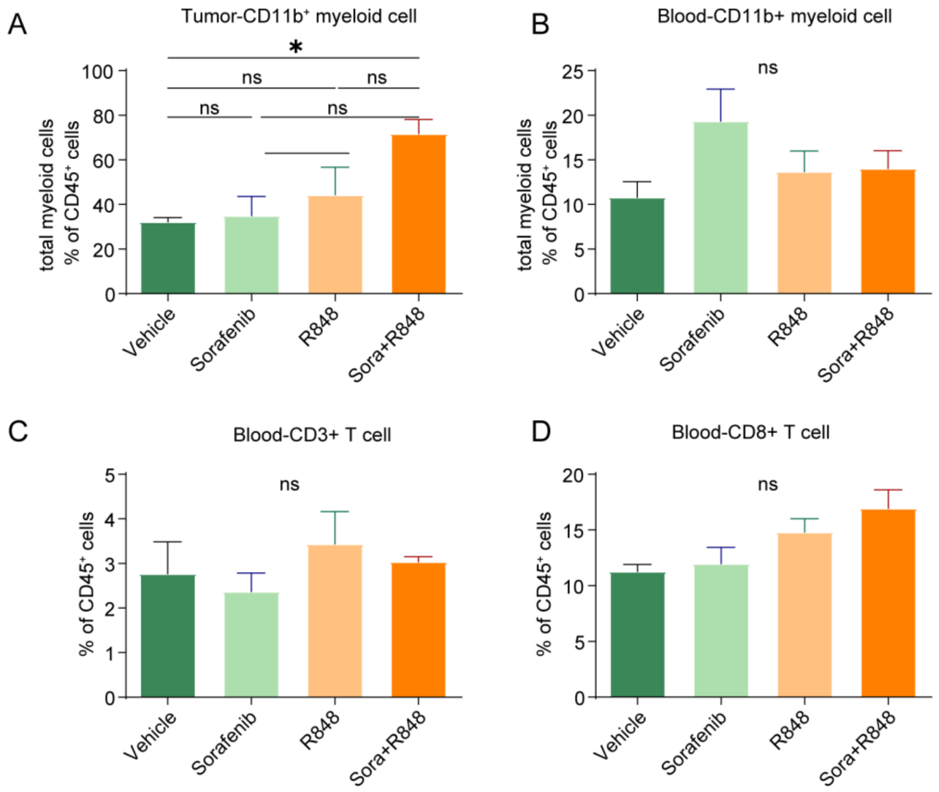

Figure S8. Immune cell populations in blood samples detected by flow cytometry analysis after sorafenib, R848 or their combination treatment. (A-B) The comparison of myeloid immune cell ratios in tumor tissues and blood samples after indicated different treatments. **(C-D)** The percentage of $CD3^+$ T cells and $CD8^+$ T cells of blood samples showing in bar graph among 4 treatment groups. Error bars represent means ± SEMs; ns: $p > 0.05$, * $p < 0.05$, ** $p < 0.01$, *** $p < 0.001$, **** $p < 0.0001$, two-way ANOVA (A-D).

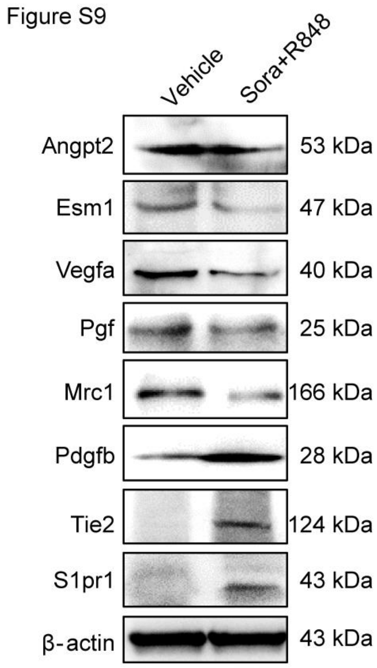

Figure S9. The western blot detection for some proteins expression including proangiogenic proteins (Angpt2, Esm1, Vegfa, Pgf, Mrc1) and vessel maturation-related proteins (Pdgfb, Tie2 and S1pr1).

Table S1. Primer sequences associated vascular stabilizing used in this study.

Mmp9	Forward-5'	GCAAGGGGCGTGTCTGGAGATTC
	Reverse-5'	GCCCACGTCGTCCACCTGGTT
Angpt2	Forward-5'	ACCGGTCAGCACCGCTACGTG
	Reverse-5'	TGCGTCAAACCACCAGCCTCCTG
Esm1	Forward-5'	TCTGCCTCCCACACAGAGCGTG
	Reverse-5'	GGAGGGCCGAGCAGCGTTC
Vegfa	Forward-5'	TGCCAAGTGGTCCCAGGCTGC
	Reverse-5'	CCTGCACAGCGCATCAGCGG
Pgf	Forward-5'	GAGGCCAGAAAGTCAGGGGGC
	Reverse-5'	ATGGGCCGACAGTAGCTGCGA
Igf1	Forward-5'	TGCTGTGTAAACGACCCGGACCT
	Reverse-5'	AGCCATAGCCTGTGGGCTTGTTGAA
Mrc1	Forward-5'	AGGCTGCCGGAAAGGCTGGAA
	Reverse-5'	GCACCTGCTCGTCCACAGTCCA
Pdgfb	Forward-5'	GGGCCCGGAGTCGGCATGAA
	Reverse-5'	AGCTCAGCCCCATCTTCATCTACGG
Tek	Forward-5'	ACGGACCATGAAGATGCGTCAACAA
	Reverse-5'	TCACATCTCCGAACAATCAGCCTGG
S1PR1	Forward-5'	GGAGAAATACCACCCCAGGC
	Reverse-5'	GGAGAGCTTTAACCTCCGGG
β-actin	Forward-5'	CACTGTCGAGTCGCGTCC

	Reverse-5'	CGCAGCGATATCGTCATCCA
